# SERPINE2-mediated activation of JAK2/STAT3 facilitates NRF2 nuclear translocation and GCLC transcription to confer ferroptosis resistance and lenvatinib resistance in hepatocellular carcinoma

**DOI:** 10.1186/s13046-026-03746-y

**Published:** 2026-06-06

**Authors:** Kan Liu, Shenglan Huang, Yongkang Xu, Ye Mao, Ning Chen, Yixin Yuan, Qinwen Xiao, Shumin Fu, Jianbing Wu

**Affiliations:** Department of Oncology, The Second Affiliated Hospital, Jiangxi Medical College, Nanchang University, No. 1, Minde Road, Donghu District, Nanchang, Jiangxi Province P.R. China

**Keywords:** Hepatocellular carcinoma, Lenvatinib resistance, Ferroptosis, SERPINE2, STAT3, GCLC

## Abstract

**Background:**

Acquired resistance remains a major obstacle in molecular-targeted therapy for advanced hepatocellular carcinoma (HCC). This study aimed to elucidate the role and underlying mechanisms of SERPINE2 in mediating lenvatinib resistance by suppressing ferroptosis.

**Methods:**

We analyzed SERPINE2 expression and its clinical prognostic relevance using public databases (TCGA) and clinical samples (tumor tissues and serum) from patients with HCC treated with lenvatinib. By establishing hypoxic and acquired lenvatinib-resistant HCC cell models, combined with subcutaneous xenograft and orthotopic liver cancer mouse models, we systematically evaluated the functional role of SERPINE2 in tumor proliferation, apoptosis, ferroptosis, and lenvatinib resistance in vitro and in vivo. Mechanistic insights were obtained through transcriptome sequencing (RNA-seq), quantitative proteomics, co-immunoprecipitation (Co-IP), nuclear-cytoplasmic fractionation, and measurement of key ferroptosis indicators (lipid ROS, mitochondrial membrane potential, and transmission electron microscopy). Finally, we assessed the therapeutic potential of targeted interventions combining a JAK inhibitor (Ruxolitinib) or NRF2 inhibitor (ML385) with lenvatinib to overcome resistance in vitro and in vivo.

**Results:**

SERPINE2 was markedly upregulated in hypoxic and lenvatinib-resistant HCC models and clinical tissues, correlating with a poor prognosis. Mechanistically, SERPINE2 activates the JAK2/STAT3 signaling pathway, promotes the STAT3-NRF2 interaction, and facilitates NRF2 nuclear translocation. This upregulates the antioxidant enzyme GCLC, which strengthens glutathione synthesis, inhibits lipid peroxidation and ferroptosis, and ultimately confers lenvatinib resistance. Inhibiting SERPINE2, STAT3, or GCLC restored ferroptosis and re-sensitized HCC cells to lenvatinib. Furthermore, the combination treatments exhibited significant synergistic antitumor effects in vitro and in vivo. Clinically, elevated SERPINE2 levels positively correlated with GCLC, predicted worse survival outcomes, and effectively distinguished lenvatinib-resistant patients.

**Conclusions:**

This study established the SERPINE2–JAK2/STAT3–NRF2–GCLC signaling axis as a key mechanism driving ferroptosis resistance and lenvatinib treatment failure. Targeting this axis provides a novel therapeutic strategy for overcoming lenvatinib resistance in HCC.

**Supplementary Information:**

The online version contains supplementary material available at 10.1186/s13046-026-03746-y.

## Introduction

Hepatocellular carcinoma (HCC) is the most common type of primary liver cancer, accounting for approximately 80–90% of all cases, and is the third leading cause of cancer-related mortality worldwide [1]. HCC is characterized by high malignancy, an insidious onset, and rapid disease progression. More than 70% of the patients are diagnosed at intermediate or advanced stages, rendering them ineligible for curative treatment. Consequently, therapeutic options are extremely limited, and the 5-year overall survival rate remains < 18% [2, 3]. Although anti-angiogenic targeted therapy combined with immunotherapy has rapidly emerged as a major treatment strategy for advanced malignancies, a substantial proportion of HCC patients are unable to tolerate combination regimens due to immune-related adverse events or contraindications [4, 5]. As a result, multi-target tyrosine kinase inhibitors (TKIs) remain the cornerstone of systemic therapy for advanced HCC. Lenvatinib, a multi-target tyrosine kinase receptor inhibitor, has been widely adopted as a first-line treatment for advanced HCC in clinical practice. However, real-world clinical data indicate that the median overall survival of patients treated with lenvatinib is approximately one year, with an objective response rate (ORR) of only 20–25%. The limited clinical efficacy of lenvatinib is largely attributed to its rapid development of drug resistance, which severely compromises its long-term therapeutic benefits [6, 7]. Previous studies have reported that more than 60% of patients with HCC develop resistance to lenvatinib within one year of treatment, and only a small subset of patients achieve sustained clinical benefit [8, 9]. Therefore, there is an urgent need to elucidate the molecular mechanisms underlying lenvatinib resistance and to identify novel therapeutic targets for the development of effective anti-resistance strategies in HCC.

The tumor microenvironment plays a critical role in tumor initiation, progression, and therapeutic response. Among its components, intratumoral hypoxia is a hallmark feature of HCC, primarily resulting from aberrant vascular architecture and rapid tumor growth, which leads to insufficient oxygen supply. Hypoxic conditions profoundly reprogram tumor cell metabolism, redox homeostasis, and survival-related signaling pathways, thereby promoting tumor aggressiveness and resistance to anticancer therapies [10–12]. Ferroptosis is a recently identified form of regulated cell death that is distinct from apoptosis, necrosis, and autophagy and is characterized by the iron-dependent accumulation of lipid peroxides [13]. Increasing evidence indicates that ferroptosis plays a pivotal role in tumor development and therapeutic response and is closely associated with the efficacy of various anticancer treatments, including targeted therapy, chemotherapy, and immunotherapy. Notably, ferroptosis resistance has emerged as a critical mechanism by which tumor cells acquire resistance to targeted therapies [14]. During anticancer treatment, many targeted agents exert their antitumor effects by inducing intracellular oxidative stress and lipid peroxidation, thereby directly or indirectly activating the ferroptotic pathway. In contrast, tumor cells can survive under therapeutic pressure by suppressing ferroptosis through the upregulation of glutathione synthesis, activation of lipid peroxide–detoxifying systems, and maintenance of cellular redox homeostasis, ultimately leading to the development of drug-resistant phenotypes [15]. Glutamate–cysteine ligase catalytic subunit (GCLC), the rate-limiting enzyme in glutathione biosynthesis, plays a central role in maintaining antioxidant defenses and suppressing lipid peroxidation. The upregulation of GCLC enhances intracellular glutathione reserves and sustains the activity of glutathione peroxidase 4 (GPX4), thereby effectively inhibiting ferroptosis. Tumor cells can acquire ferroptosis tolerance under therapeutic stress by reinforcing GCLC-mediated antioxidant capacity [16, 17]. However, the regulatory mechanisms governing GCLC expression in hypoxia-associated targeted therapy resistance remain largely unclear.

Serine protease inhibitor E family member 2 (SERPINE2), also known as protease nexin-1 (PN-1), exhibits antiserine protease activity against thrombin, urokinase, and plasminogen [18]. Previous studies have demonstrated that SERPINE2 can inhibit epidermal growth factor receptor (EGFR) degradation and plays an important role in the metastatic progression of HCC [19]. However, whether SERPINE2 contributes to lenvatinib resistance in HCC has not yet been thoroughly investigated. In the present study, we found that SERPINE2 was markedly upregulated in hypoxic HCC cells, lenvatinib-resistant HCC cells, and tumor tissues. Elevated SERPINE2 expression promotes transcriptional upregulation of GCLC, thereby suppressing ferroptosis in HCC cells. Mechanistically, SERPINE2 activates the JAK2/STAT3 signaling pathway, and activated STAT3 physically interacts with nuclear factor erythroid 2–related factor 2 (NRF2), facilitating its nuclear translocation and enhancing NRF2-mediated GCLC transcription. This signaling axis reinforces cellular antioxidant capacity, inhibits lipid peroxidation, and ultimately blocks ferroptotic cell death, leading to the development of lenvatinib resistance in HCC cells. Furthermore, we elucidated the clinical significance of SERPINE2 and GCLC in HCC and demonstrated that elevated expression levels are closely associated with poor patient prognosis and reduced responsiveness to lenvatinib treatment. Using in vivo models, we further explored the therapeutic potential of a JAK pathway inhibitor (Ruxolitinib) and selective NRF2 inhibitor (ML385) to overcome lenvatinib resistance in HCC. Collectively, our findings identified a previously unrecognized SERPINE2–JAK2/STAT3–NRF2–GCLC signaling axis that mediates ferroptosis suppression and targeted therapy resistance, providing novel mechanistic insights and potential therapeutic targets for overcoming lenvatinib resistance in hepatocellular carcinoma.

## Materials and methods

### Clinical sample collection and follow-up

Forty patients with HCC who received preoperative lenvatinib treatment at the Second Affiliated Hospital of Nanchang University between January 2021 and December 2024 were enrolled in this study. Therapeutic response to lenvatinib was evaluated according to the modified Response Evaluation Criteria in Solid Tumors (mRECIST). Based on treatment outcomes, the patients were classified into two groups: lenvatinib-sensitive (including complete response [CR] and partial response [PR]) and lenvatinib-resistant (progressive disease [PD]). All enrolled patients had complete baseline and follow-up clinical data (Table S1, Supporting Information). Tumor tissues and paired adjacent non-tumorous liver tissues were collected from each patient. One portion of each specimen was fixed in formalin and embedded in paraffin for subsequent IHC and IF analyses to evaluate gene expression patterns. The remaining portion was snap-frozen and stored at − 80 °C for subsequent protein and RNA extraction to validate gene expression levels. Overall survival (OS) was defined as the interval from the initiation of lenvatinib treatment to death from any cause. In addition, peripheral blood samples were collected from 216 patients with HCC who received lenvatinib treatment during the same period(Table S2, Supporting Information), including samples obtained at the time of best response and after confirmation of progressive disease. Serum SERPINE2 concentrations were subsequently measured using ELISA kits, and archived paraffin-embedded tumor tissue specimens were obtained from the Department of Pathology of the Second Affiliated Hospital of Nanchang University. After screening, 182 eligible paraffin-embedded tumor samples from lenvatinib-treated HCC patients were included for further analysis (Table S3, Supporting Information). Immunofluorescence staining was performed to assess SERPINE2 and GCLC expression, and patients were stratified into subgroups based on median expression levels, which was conducted in accordance with the principles of the Declaration of Helsinki and was approved by the Ethics Committee of the Second Affiliated Hospital of Nanchang University. Written informed consent was obtained from all participants prior to enrollment.

### Cell culture

Human hepatocellular carcinoma cell lines Huh7 and PLC/PRF/5 were obtained from the Cell Bank of the Chinese Academy of Sciences (Shanghai, China). The murine hepatocellular carcinoma cell line Hepa1-6 was purchased from Boster (China; CX0019). All cell lines were cultured in Dulbecco’s Modified Eagle’s medium (DMEM; Solarbio, Beijing, China; 11995) supplemented with 10% fetal bovine serum (FBS; ExCell Bio, Suzhou, China; FSP500) and 1% penicillin–streptomycin. The cells were maintained in a humidified incubator at 37 °C and 5% CO₂.

### Establishment of acquired lenvatinib-resistant and hypoxic HCC cell models

Acquired lenvatinib-resistant HCC cell models were generated using a stepwise dose escalation strategy. The human HCC cell lines Huh7 and PLC/PRF/5 were used as parental cells. Lenvatinib was dissolved in dimethyl sulfoxide (DMSO) to prepare a stock solution and gradually introduced into the culture medium. During the induction of resistance, the cells were initially exposed to a low concentration of lenvatinib. Once cells regained stable proliferation, as evidenced by normal morphology and growth rates comparable to those of parental cells, the drug concentration increased incrementally. The lenvatinib concentration was increased every two passages until a predetermined target concentration was reached. Cells capable of sustained proliferation under high lenvatinib concentrations were defined as lenvatinib-resistant and designated Huh7-LR and PLC/PRF/5-LR, respectively. The IC₅₀ was determined by cell viability assays and compared with that of parental cells to confirm successful establishment of resistant models. Resistant cell lines were maintained in a medium containing a maintenance dose of lenvatinib, and all functional experiments were performed after culturing the cells in drug-free medium for 48 h to eliminate acute drug effects.

The lenvatinib-resistant Hepa1-6 cell line was established based on previously reported methods, with modifications [20]. Briefly, Hepa1-6 cells (5 × 10⁶ cells per mouse) were subcutaneously injected into the right dorsal flank of 4-week-old male C57BL/6 mice to establish xenograft tumors. When tumor volumes reached approximately 100 mm³, mice were treated with lenvatinib at a dose of 10 mg/kg/day. After 28 consecutive days of treatment, the mice were euthanized, and the largest tumors were excised and cut into fragments of approximately 1 mm³. These tumor fragments were subsequently re-implanted subcutaneously into a new generation of 4-week-old male C57BL/6 mice, followed by repeated lenvatinib treatment and tumor harvesting. This in vivo selection procedure was repeated for three passages to maintain a continuous drug selection pressure. Tumor tissues obtained from the final passage were dissociated and cultured to establish a stable lenvatinib-resistant Hepa1-6 cell line for subsequent in vitro and in vivo experiments.

To mimic the hypoxic tumor microenvironment in vivo, hypoxic HCC cell models were established by culturing HCC cells under hypoxic conditions. Specifically, cells were incubated in a hypoxia chamber with a controlled gas composition of 1% O₂, 5% CO₂, and 94% N₂ at 37 °C for 48 h. Cells cultured under normoxic conditions (21% O₂, 5% CO₂) served as controls. The successful establishment of the hypoxic model was verified by assessing the expression levels of hypoxia-inducible factor-1α (HIF-1α).

### Animal experiments

Four-week-old male wild-type C57BL/6 and BALB/c nude mice were purchased from Jiangsu Jicui Yaokang Biotechnology Co., Ltd. (animal production license number: SCXK (Su) 2023-009). All mice were housed under specific pathogen-free (SPF) conditions, maintained at a temperature of 22 °C and a relative humidity of 40–60%, with a 12-h light/12-h dark cycle. Animals were provided with food and water ad libitum and were euthanized by carbon dioxide inhalation when tumor burden and overall health status reached predefined humane endpoints. All animal experimental procedures were approved by the Animal Welfare and Ethics Committee of Leyou Biotechnology (Nanchang, China) (approval number: RYE2025082202) and conducted in strict accordance with the ARRIVE guidelines.

### Establishment of subcutaneous xenograft models and treatment protocol

Four-week-old male BALB/c nude mice were housed under SPF conditions with free access to food and water. Huh7-LR, Huh7-LR (shSERPINE2), PLC/PRF/5, and PLC/PRF/5 (OE-SERPINE2) cells in the logarithmic growth phase were harvested, resuspended in antibiotic-free DMEM, and subcutaneously injected into the right axillary region of mice at a density of 5 × 10⁶ cells per mouse.Tumor growth was monitored regularly after inoculation. When tumor volumes reached approximately 80–100 mm³, the mice were randomized to receive different treatments according to the experimental design, including control, control combined with lenvatinib, treatment alone, or treatment combined with lenvatinib. Lenvatinib was administered by oral gavage at a dose of 10 mg/kg/day for three consecutive weeks. Tumor length (L) and width (W) were measured every 2–3 days using calipers and tumor volume was calculated using the formula V = (L × W²)/2. Body weight and general health status were recorded throughout the treatment period, at which time the mice were euthanized and tumor tissues were excised, weighed, and fixed for subsequent analyses.

### Establishment of orthotopic hepatocellular carcinoma model and treatment protocol

Four-week-old male C57BL/6 mice were housed under SPF conditions with free access to food and water. Lenvatinib-resistant Hepa1-6 cells in the logarithmic growth phase were harvested, resuspended in serum-free medium, and adjusted to a concentration of 1 × 10⁷ cells/mL. Mice were anesthetized and subjected to aseptic surgery. A midline abdominal incision was made to expose the left hepatic lobe gently. Subsequently, 1 × 10⁶ tumor cells in a volume of 50 µL were slowly injected into the parenchyma of the left liver lobe using a microsyringe. After the injection, the puncture site was gently compressed using a sterile cotton swab to prevent cell leakage. Upon confirmation of hemostasis, the liver was repositioned, and the abdominal wall and skin were closed layer-by-layer, after which the mice were allowed to recover in a temperature-controlled environment and were closely monitored for general condition. Seven days after tumor implantation, in vivo imaging was performed and mice with comparable tumor burdens were randomly assigned to different treatment groups. According to the experimental design, mice received PBS, lenvatinib monotherapy, ruxolitinib or ML385 monotherapy, or a combination of lenvatinib plus ruxolitinib or ML385, as shown in Fig. 9B. During the treatment period, body weight, physical condition, and survival status were monitored regularly, at which point the mice were euthanized, and the livers were harvested to record the number and volume of the tumor nodules. Portions of the tumor tissues were collected for the assessment of ferroptosis-related indicators, while the remaining tissues were fixed for histological examination and immunohistochemical analyses. The remaining mice were followed up until the observation endpoint for survival analysis.

### RNA sequencing (RNA-seq) analysis

Total RNA was extracted using TRIzol reagent according to the manufacturer’s instructions. RNA concentration, purity, and integrity were assessed, and only samples that met the quality control criteria were used for subsequent experiments. Poly(A)+ mRNA was enriched using oligo(dT) magnetic beads, fragmented, and reverse-transcribed to generate cDNA, followed by the construction of strand-specific sequencing libraries. After library quality control, paired-end sequencing (PE150) was performed using the Illumina NovaSeq 6000 platform. RNA sequencing and bioinformatic analyses were conducted by LC-Bio Technology Co., Ltd. (Hangzhou, China). DEGs were identified using the criteria of *P* < 0.05 and |log2 fold change| > 1.

### Astral DIA-based proteomic analysis

Proteomic profiling was performed using astral data-independent acquisition (DIA) strategy. Proteins were extracted using urea-containing lysis buffer, quantified, and subjected to quality control, followed by reduction and alkylation. Proteins were digested with trypsin and the resulting peptides were desalted by solid-phase extraction. The peptides were further fractionated by high-pH reversed-phase liquid chromatography and combined into multiple fractions for subsequent analysis. The peptide samples were separated using a (nNano-LC) system and analyzed on a Thermo Scientific Astral mass spectrometer operating in DIA mode. Raw DIA data were processed using DIA-NN software (version 1.9.1) for protein identification and quantification, with spectral libraries generated using the DirectDIA approach. Search parameters included Trypsin/P digestion with up to two missed cleavages, carbamidomethylation of cysteine as a fixed modification, and oxidation of methionine and protein N-terminal acetylation as variable modifications. A target–decoy strategy was applied, with false discovery rates (FDRs) set to < 0.01 at both the peptide and protein levels. Proteomic sequencing and integrative analyses were performed by LC-Bio Technology Co. Ltd. (Hangzhou, China).

### Astral-Zoom-DIA quantitative proteomic analysis

Astral-Zoom-DIA-based quantitative proteomics were conducted using a data-independent acquisition strategy. After enzymatic digestion, the peptide samples were separated using a Thermo Scientific Vanquish Neo UHPLC system and loaded onto an ES906 HPLC column, followed by analysis on an Orbitrap™ Astral™ Zoom mass spectrometer (LC-Bio Technology, Hangzhou, China) operating in positive ion mode. Raw DIA data were analyzed using DIA-NN software (version 1.9.2) for protein identification and quantification, with spectral libraries constructed using the DirectDIA mode. Search parameters were set as follows: trypsin/P digestion with a maximum of two missed cleavages, carbamidomethylation of cysteine as a fixed modification, and oxidation of methionine and protein N-terminal acetylation as variable modifications. The target–decoy strategy was employed to control false positives at both peptide and protein levels, with FDR thresholds of < 0.01 at both the peptide and protein levels. All sequencing procedures and data analyses were performed by LC-Bio Technology Co. Ltd. (Hangzhou, China).

### siRNA-mediated gene silencing

Small interfering RNAs (siRNAs) targeting the indicated genes were purchased from HanHeng Biology (Shanghai, China), and the corresponding sequences are listed in Table S4 (Supporting Information). Cells were seeded in 6-well plates 24 h prior to transfection to reach approximately 40%–60% confluence at the time of transfection. siRNA transfection was performed using Lipofectamine 3000 reagent (Thermo Fisher Scientific) according to the manufacturer’s instructions. Briefly, siRNAs and Lipofectamine 3000 were separately diluted in Opti-DMEM serum-free medium, mixed gently, and incubated at room temperature for 10–15 min to form transfection complexes, which were added dropwise to the cells. After 6–8 h, the medium was replaced with a fresh complete culture medium. Cells transfected with a non-targeting siRNA (si-NC) served as negative controls. Gene silencing efficiency was evaluated by RT–qPCR at 48 h and western blotting at 72 h post-transfection. Subsequent functional assays were performed at the indicated time points after transfection.

### Lentiviral-mediated stable knockdown cell line generation

Stable knockdown cell lines were generated using lentiviral vectors encoding short hairpin RNAs (shRNAs) targeting the indicated genes (HanHeng Biology and GeneChem, Shanghai, China; sequences are listed in Table S4,Supporting Information. shRNA constructs and a non-targeting control (sh-NC) were cloned into the lentiviral expression vectors. Target cells were seeded one day before infection to achieve 30%–50% confluence. Cells were infected with lentiviral supernatants in the presence of polybrene (4 µg/mL) to enhance transduction efficiency. After 24 h, the medium was replaced with a fresh complete medium. At 72 h post-infection, puromycin (MCE, Shanghai, China; HY-B1743A) was added for selection until all uninfected control cells were eliminated. Knockdown efficiency was confirmed by RT–qPCR and western blotting. All subsequent experiments were conducted using stable cell lines with confirmed knockdown efficiency.

### Nuclear and cytoplasmic protein extraction

Nuclear and cytoplasmic protein fractions were prepared using a Nuclear and Cytoplasmic Protein Extraction Kit (Beyotime, Shanghai, China; P0028), according to the manufacturer’s instructions. Briefly, the cells were harvested after the indicated treatments, washed twice with ice-cold PBS, and resuspended in pre-chilled cytoplasmic lysis buffer supplemented with protease and phosphatase inhibitors. After incubation on ice for 10–15 min, the samples were briefly vortexed and centrifuged to separate the cytoplasmic supernatant from the nuclear pellet. The nuclear pellet was lysed in nuclear extraction buffer on ice with intermittent vortexing, followed by high-speed centrifugation at 4 °C. The resulting supernatant was collected as the nuclear protein fraction and used for subsequent western blot analysis.

### Western blotting

Cells or tissue samples were lysed in RIPA buffer containing protease and phosphatase inhibitors (Beyotime, Shanghai, China; P0013B) on ice for 30 min. Lysates were centrifuged at 12,000 × g for 15 min at 4 °C and the supernatants were collected. Protein concentrations were determined using a BCA Protein Assay kit (Elabscience, Wuhan, China; E-BC-K318-M). Equal amounts of protein were mixed with 6× SDS-PAGE loading buffer, denatured at 95 °C for 5 min, and separated by SDS-PAGE. The proteins were transferred onto PVDF membranes (Millipore, USA; ISEQ00010). Membranes were blocked with 5% non-fat milk or 5% bovine serum albumin (BSA) at room temperature for 1 h and incubated with primary antibodies (Table S5, Supporting Information) at 4 °C overnight. After washing with TBST, membranes were incubated with HRP-conjugated secondary antibodies for 1 h at room temperature. Protein bands were visualized using enhanced chemiluminescence (ECL) reagents and imaged using the Bio-Rad imaging system. Band intensities were quantified using ImageJ software and normalized to the internal controls.

### RT–qPCR analysis

Total RNA was extracted using an RNA extraction kit (TransGen Biotech, Beijing, China; ER101-01). Equal amounts of RNA were reverse transcribed into cDNA (Table S6, Supporting Information) using a reverse transcription kit (TransGen Biotech; AE311-02). Quantitative PCR was performed using SYBR Green Master Mix (Takara, Beijing, China; RR820A) on a Bio-Rad Real-Time PCR system. The PCR conditions were as follows: 95 °C for 30 s, followed by 40 cycles of 95 °C for 5–10 s and 60 °C for 30 s. Melt curve analysis was conducted to verify the amplification specificity. GAPDH was used as an internal control, and relative gene expression levels were calculated using the 2^−ΔΔCt method.

### CCK-8 cell proliferation assay

Cell proliferation was assessed using a Cell Counting Kit-8 (CCK-8; Uelandy, Suzhou, China; C6005M). Briefly, the cells were trypsinized, counted, and seeded into 96-well plates at the indicated densities in 100µL of complete culture medium per well. After cell attachment, the CCK-8 working solution (10µL per well) was added at the indicated time points (0, 24, 48, and 72 h), followed by incubation at 37 °C for 2 h. The absorbance was measured at 450 nm (OD450) using a microplate reader. Each experimental condition was performed with 3–5 replicate wells, and the background absorbance from the blank wells was subtracted. The cell viability was expressed as the OD450 value.

### IC₅₀ determination

The IC₅₀ of lenvatinib was determined using the CCK-8 assay. The cells were seeded into 96-well plates and allowed to adhere overnight. The cells were then treated with a series of increasing concentrations of lenvatinib (0, 2.5, 5, 7.5, 10, 15, 20, 30, 40, 50, 60, and 70µM) for 48 h. CCK-8 working solution (10µL per well) was added, followed by incubation at 37 °C for 2 h, and the absorbance at 450 nm (OD450) was measured using a microplate reader. Cell viability in the untreated or vehicle-treated groups was defined as 100%, and the relative survival rate at each drug concentration was calculated accordingly. IC₅₀ values were calculated by nonlinear regression analysis using GraphPad Prism software.

### Colony formation assay

Cells were harvested, counted, and seeded at a low density into 6-well plates (typically 1,000 cells per well). After gentle agitation to ensure even distribution, the plates were incubated at 37 °C in a humidified atmosphere containing 5% CO₂. During the culture period, the medium was replaced every 2–3 d. For drug treatment experiments, cells were allowed to adhere before the addition of the indicated concentrations of drugs, and the medium was refreshed with the drugs at designated intervals. The cells were cultured until visible colonies were formed. The culture medium was removed at the end of the experiment and the cells were gently washed with PBS, fixed with 4% paraformaldehyde for 15 min, and stained with 0.1% crystal violet for 10 min. Excess stain was removed by rinsing with distilled water and the plates were air-dried. Images were captured, and the number and/or area of colonies was quantified using ImageJ software.

### EdU cell proliferation assay

Cells were seeded into 96-well plates and allowed to adhere until they reached appropriate confluence. Cell proliferation was assessed using an EdU assay kit (YaMei Biotechnology, Shanghai, China; CX003) according to the manufacturer’s instructions. Briefly, the cells were incubated with EdU working solution for 2 h. After incubation, the medium was removed, and the cells were washed with PBS, fixed with 4% paraformaldehyde for 15 min, and permeabilized with 0.2%–0.5% Triton X-100 for 10–15 min. Subsequently, Click-iT reaction was performed to label EdU-positive cells, followed by nuclear counterstaining with DAPI. Fluorescence images were captured using a fluorescence microscope, and the proportion of EdU-positive cells was quantified using ImageJ or a similar image analysis software.

### Flow cytometric analysis of apoptosis

Cells subjected to the indicated treatments were collected, including adherent and floating cells in the culture supernatant. After washing twice with PBS, the cells were resuspended in 1× Binding Buffer at a density of approximately 1–5 × 10 cells per tube. Apoptosis was assessed using an Annexin V-FITC/PI Apoptosis Detection Kit (Uelandy, Suzhou, China; F6012L) according to the manufacturer’s instructions. Briefly, Annexin V-FITC and propidium iodide (PI) were added to the cell suspension, followed by incubation for 10 min at room temperature in the dark. After incubation, an appropriate volume of Binding Buffer was added, and the samples were analyzed within 1 h using a flow cytometer (CytoFLEX, Beckman Coulter, USA). Data were analyzed using the FlowJo software. Cells negative for both Annexin V and PI (Annexin V⁻/PI⁻) were considered viable cells, Annexin V–positive and PI-negative cells (Annexin V⁺/PI⁻) were defined as early apoptotic cells, and Annexin V–positive and PI-positive cells (Annexin V⁺/PI⁺) were defined as late apoptotic or necrotic cells.

### Immunohistochemical (IHC) analysis

Tumor tissues and liver specimens were fixed in 4% paraformaldehyde, paraffin-embedded, and sectioned. Tissue sections were deparaffinized in xylene and rehydrated using graded ethanol solutions, followed by heat-induced antigen retrieval using citrate buffer (pH 6.0) or EDTA buffer (pH 9.0). Endogenous peroxidase activity was blocked by incubation with 3% hydrogen peroxide for 10 min at room temperature, and nonspecific binding was blocked with 5% BSA or normal goat serum for 30 min at room temperature. The sections were incubated overnight with primary antibodies at 4 °C. After washing with TBST, the sections were incubated with appropriate HRP-conjugated secondary antibodies for 60 min at room temperature. Signal detection was performed using DAB substrate, followed by counterstaining with hematoxylin. The sections were then dehydrated, cleared, and mounted. Images were captured using a light microscope. The IHC results were evaluated using the semi-quantitative immunohistochemical scoring (IHC score) method.

### Immunofluorescence (IF) staining

For immunofluorescence staining, the cells were seeded onto glass coverslips and subjected to the indicated treatments upon reaching the appropriate confluence. Cells were washed twice with PBS and fixed with 4% paraformaldehyde at room temperature for 15–20 min, followed by permeabilization with 0.2%–0.5% Triton X-100 for 10–15 min. After blocking with 5% bovine serum albumin (BSA) at room temperature for 30–60 min, the cells were incubated with primary antibodies at 4 °C overnight. The following day, the cells were washed thrice with PBS and incubated with the appropriate fluorophore-conjugated secondary antibodies for 1 h at room temperature in the dark. Nuclei were counterstained with DAPI or Hoechst, coverslips were mounted after washing, and samples were fixed in 4% paraformaldehyde, paraffin-embedded, and sectioned. The sections were deparaffinized in xylene and rehydrated using graded ethanol, followed by antigen retrieval. After cooling to room temperature and washing with PBS, the cells were stained as described above. Fluorescence images were acquired using a fluorescence microscope.

### Transmission Electron Microscopy (TEM) analysis of mitochondria

The cells were collected and fixed with 2.5% glutaraldehyde at 4 °C for 24 h, washed with buffer, and post-fixed with 1% osmium tetroxide for 1 h. After fixation, the samples were sequentially dehydrated using a graded ethanol series, infiltrated, and embedded in epoxy resin 812. Ultrathin sections (~ 50 nm) were prepared using an ultramicrotome and stained with uranyl acetate for 15 min and lead citrate for 1 min. The ultrastructural morphology of the mitochondria was examined, and images were captured using a transmission electron microscope (HITACHI HT7800, Hitachi, Japan).

### Measurement of mitochondrial membrane potential (ΔΨm)

Mitochondrial membrane potential (ΔΨm) was assessed using a JC-1 Mitochondrial Membrane Potential Assay Kit (Beyotime, Shanghai, China; C2006) according to the manufacturer’s instructions. Briefly, cells were seeded in 6-well plates at a density of 6 × 10 cells per well. After cell attachment and the indicated treatments, the cells were incubated with 500 µL of JC-1 working solution for 20 min at 37 °C. Following incubation, JC-1 fluorescence was detected at excitation/emission wavelengths of 514/529 nm for JC-1 monomers and 585/590 nm for JC-1 aggregates, using a confocal laser scanning microscope (Nikon C2+, Japan) and a flow cytometer (CytoFLEX, Beckman Coulter, USA). Changes in mitochondrial membrane potential were evaluated by calculating the ratio of JC-1 aggregates to JC-1 monomer fluorescence intensity.

### Detection of lipid Reactive Oxygen Species (lipid ROS)

Cells were seeded into 48-well plates at a density of 3 × 10 cells/well. After the indicated treatments, the cells were incubated with 5 µM BODIPY 581/591 C11 probe (Beyotime, Shanghai, China; S0043S) at 37 °C for 1 h in the dark. Following incubation, fluorescence images were captured using a confocal laser scanning microscope (Nikon C2+, Japan) to assess the intracellular lipid reactive oxygen species levels.

### MDA and GSH/GSSG

Cells were collected after the indicated treatments and lysed according to the standard procedures. The levels of malondialdehyde (MDA) were measured using a commercial MDA assay kit (MCE, Shanghai, China; HY-K0319), and reduced/oxidized glutathione (GSH/GSSG) levels were determined using a GSH/GSSG assay kit (MCE, Shanghai, China; HY-K0311), following the manufacturer’s instructions. The MDA content was used as an indicator of lipid peroxidation and oxidative damage, while the GSH/GSSG ratio was calculated to reflect the cellular redox status. All measurements were normalized to the total protein concentration and comparative analyses were performed between the experimental groups.

### Measurement of intracellular ferrous iron (Fe²⁺)

Fe²⁺ levels were detected using a FerroOrange live-cell fluorescent probe (MCE, Shanghai, China; HY-K0322). The FerroOrange probe was first prepared in DMSO and then diluted to the working concentration according to the manufacturer’s instructions. After the indicated treatments, the cells were seeded into confocal culture dishes at a density of 1 × 10⁴ cells per well and incubated with 250 µL of FerroOrange working solution at 37 °C for 60 min in the dark. Following incubation, the nuclei were counterstained with Hoechst dye (Uelandy, Suzhou, China; H4079). Fluorescence images were acquired using a confocal laser-scanning microscope (Nikon C2+, Japan). The intracellular fluorescence intensity of FerroOrange was detected at excitation/emission wavelengths of 543/580 nm and used to evaluate intracellular Fe²⁺ accumulation.

### ELISA

Peripheral blood samples were collected into coagulation tubes and allowed to clot at room temperature, followed by centrifugation at 3,000 rpm for 10 min to separate the serum. Serum samples were aliquoted and stored at − 80 °C until use, avoiding repeated freeze–thaw cycles. Serum SERPINE2 protein levels were measured using a commercial ELISA kit (Huamei Biotechnology, Wuhan, China; CSB-EL021082HU), and serum alanine aminotransferase (ALT) levels were determined using an ALT ELISA kit (Elabscience, Wuhan, China; E-BC-K325-M) according to the manufacturer’s instructions. Briefly, standards and appropriately diluted serum samples were added to the pre-coated plates and incubated. After washing, detection antibodies and HRP conjugates were added sequentially. Color development was achieved using a substrate solution and was terminated with a stop solution. The absorbance was measured at 450 nm using a microplate reader. The concentrations of SERPINE2 and ALT in the serum samples were calculated based on standard curves.

### In vivo bioluminescence imaging of mouse liver tumors

To dynamically monitor the tumor burden in the orthotopic liver cancer mouse model, in vivo bioluminescence imaging was performed at the indicated time points. Lenvatinib-resistant Hepa1-6 cells (Hepa1-6-LR-Luc) were used to establish an orthotopic liver tumor model. On the day of imaging, the mice were anesthetized with isoflurane inhalation and intraperitoneally injected with D-luciferin (150 mg/kg, dissolved in sterile PBS). Ten minutes after injection, the mice were placed in a small animal in vivo imaging system (Clinx IVScopeEQ Capture, Clinx Science Instruments, Shanghai, China) for bioluminescence signal acquisition. Continuous anesthesia and body temperature maintenance were ensured throughout the imaging procedure. Bioluminescence data were analyzed using accompanying software. Regions of interest (ROIs) were defined over the liver area, and total photon flux (photons/s) was quantified to evaluate tumor burden and compare tumor growth among different treatment groups.

### Co-immunoprecipitation (Co-IP) assay

Cells subjected to the indicated treatments were collected and lysed on ice for 30 min using a pre-chilled IP lysis buffer (Beyotime, Shanghai, China; P0012). Cell lysates were centrifuged at 12,000 × g for 15 min at 4 °C, and supernatants were collected as total protein extracts. A portion of the supernatant was used as the input control. To reduce nonspecific binding, the remaining lysates were precleared with Protein A/G magnetic beads (MCE, Shanghai, China; HY-K0202) for 1 h at 4 °C. Subsequently, the lysates were incubated with specific primary antibodies at 4 °C with gentle rotation overnight, and an equal amount of isotype-matched IgG was used as a negative control. Protein A/G magnetic beads were then added and incubated for 2–4 h at 4 °C to capture the immune complexes. Beads were washed four to five times with cold lysis buffer, followed by elution with SDS-PAGE loading buffer and boiling at 95 °C for 5 min to dissociate the immunocomplexes. The eluted proteins were separated by SDS-PAGE, transferred to membranes, and analyzed by western blotting to examine protein–protein interactions.

### Reagents

The reagents used in this study were as follows: lenvatinib (MCE, Shanghai, China; HY-10981 A), puromycin (MCE, Shanghai, China; HY-B1743A), erastin (MCE, Shanghai, China; HY-15763), ferrostatin-1 (MCE, Shanghai, China; HY-100579), ML385 (MCE, Shanghai, China; HY-100523), ruxolitinib (MCE, Shanghai, China; HY-50856), sulfobutylether-β-cyclodextrin (SBE-β-CD; MCE, Shanghai, China; HY-17031), and D-luciferin potassium salt (MCE, Shanghai, China; HY-12591B).

### TCGA data analysis

Bioinformatic analyses were performed using LIHC from TCGA database via the Tumor Immune Estimation Resource (TIMER; https://timer.cistrome.org/) online platform. The Gene_DE and Correlation modules in TIMER were used to compare the expression levels of SERPINE2, GCLC, and STAT3 between HCC tumor tissues and normal tissues, and to evaluate expression correlations among these genes. Survival analysis was conducted using the survival module in TIMER. Patients were stratified into high- and low-expression groups according to gene expression levels. Kaplan–Meier survival curves were generated, and differences between groups were assessed using the log-rank test.

### Data analysis and statistical methods

All data are presented as mean ± standard deviation (SD). Statistical analyses were performed using GraphPad Prism version 9.5. Comparisons between two groups were conducted using a two-tailed Student’s t-test, whereas comparisons among multiple groups were performed using one-way analysis of variance (ANOVA) followed by appropriate post-hoc multiple-comparison tests. The half-maximal inhibitory concentration (IC₅₀) in drug sensitivity assays was calculated by nonlinear regression analysis using the log[inhibitor] versus response (variable slope) model. Correlation analyses were performed using Pearson’s or Spearman’s correlation test, as appropriate. Survival curves were generated using the Kaplan–Meier method and compared using the log-rank test. Receiver operating characteristic (ROC) curve analysis was applied to evaluate the predictive performance of the biomarkers, and the area under the curve (AUC) as well as the optimal cutoff value were calculated. Statistical significance was set at *P* < 0.05.

## Results

### SERPINE2 expression is markedly increased in hypoxic and lenvatinib-resistant hepatocellular carcinoma cells

**Fig. 1:**
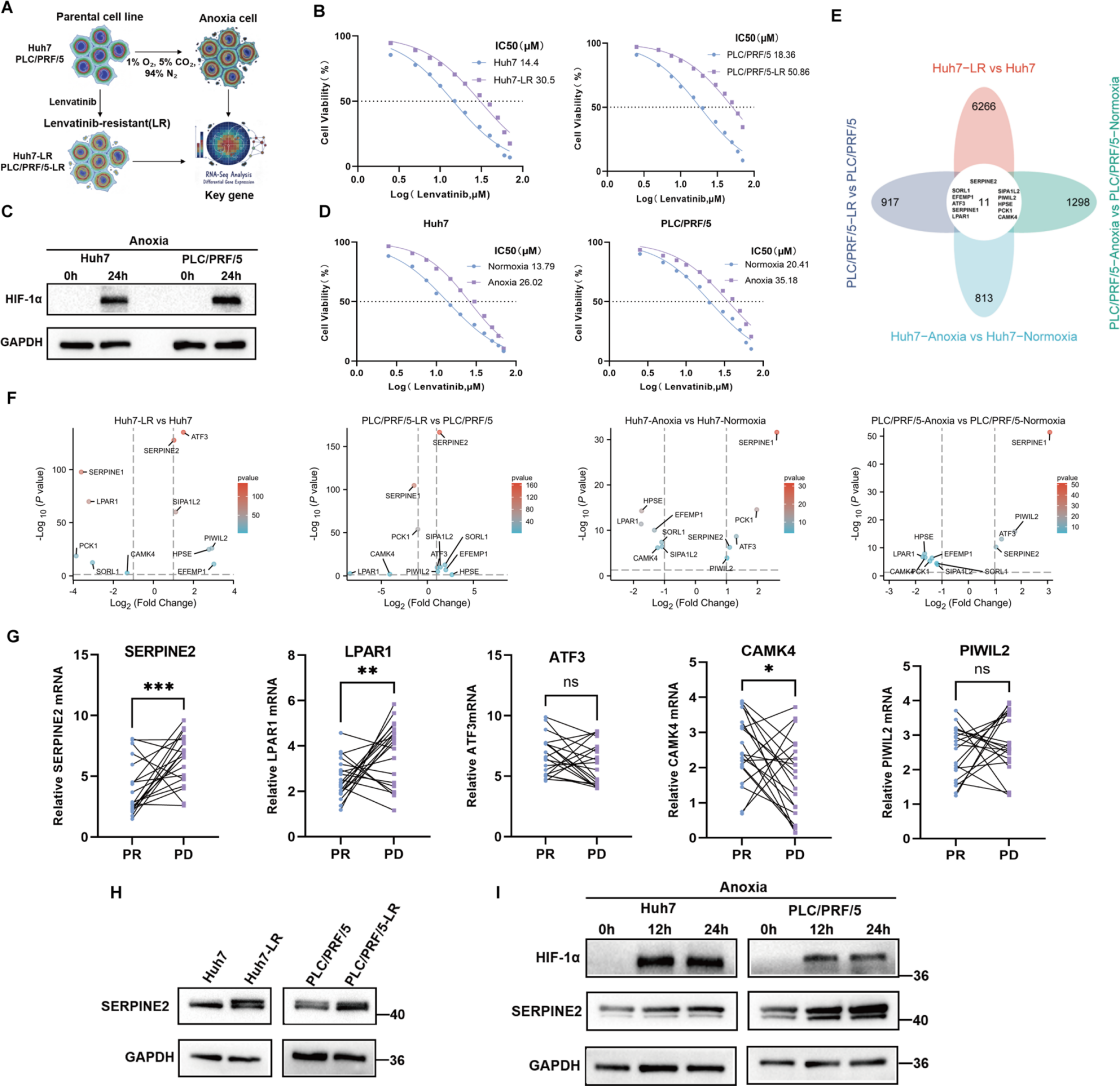
SERPINE2 is markedly upregulated in hypoxic and lenvatinib-resistant hepatocellular carcinoma. **A** Schematic illustration of the experimental workflow showing the establishment of hypoxic HCC cell models and lenvatinib-resistant (LR) cell models, followed by RNA sequencing and integrative analysis. **B** Dose–response curves of lenvatinib in parental and lenvatinib-resistant Huh7 (Huh7-LR) and PLC/PRF/5 (PLC/PRF/5-LR) cells. The IC₅₀ values were significantly increased in LR cells. **C** Western blot analysis of HIF-1α expression in HCC cells cultured under normoxic (21% O₂) or hypoxic (1% O₂) conditions, confirming successful establishment of the hypoxic cell model. GAPDH served as a loading control. **D** Comparison of lenvatinib IC₅₀ values in HCC cells under normoxic and hypoxic conditions, indicating reduced sensitivity to lenvatinib following hypoxic exposure. **E** Venn diagram showing the overlap of differentially expressed genes (DEGs) identified by RNA-seq between “lenvatinib-resistant cells vs. parental cells” and “hypoxic cells vs. normoxic cells.” **F** Volcano plot illustrating the expression patterns of the overlapping DEGs under both conditions, highlighting genes with concordant changes. **G** RT–qPCR validation of candidate genes (SERPINE2, ATF3, LPAR1, PIWIL2, and CAMK4) in tumor tissues from lenvatinib-resistant (PD) and lenvatinib-sensitive (PR) HCC patients. GAPDH was used as an internal control. **H** Western blot analysis of SERPINE2 expression in parental and lenvatinib-resistant HCC cell lines. **I** Western blot analysis showing that SERPINE2 expression was significantly increased in HCC cells cultured under hypoxic conditions compared with normoxic controls. Data are presented as mean ± SD. Statistical analyses were performed using appropriate Student’s t-tests or one-way ANOVA. Statistical significance is indicated as follows: ns, not significant; *, *p* < 0.05; **, *p* < 0.01; ***, *p* < 0.001

Tumor hypoxia is a hallmark feature of hepatocellular carcinoma (HCC) and the hypoxic microenvironment has emerged as a central driving factor in the development of acquired resistance to lenvatinib [21]. To elucidate the potential molecular mechanisms linking hypoxia to acquired lenvatinib resistance, we established hypoxic HCC cell models and lenvatinib-resistant HCC cell models, followed by transcriptome sequencing (RNA-seq) and integrated analysis to identify key genes commonly altered in both conditions. The overall experimental workflow is illustrated in Fig. 1A. Lenvatinib-resistant Huh7 (Huh7-LR) and PLC/PRF/5 (PLC/PRF/5-LR) cell lines were generated using a stepwise dose escalation strategy. Compared to their parental counterparts, lenvatinib-resistant cell lines exhibited significantly higher half-maximal inhibitory concentration (IC_50_) values for lenvatinib (Fig. 1B). The validity of the lenvatinib-resistant models was further confirmed by functional assays including flow cytometric apoptosis analysis (Figure S1A, Supporting Information), colony formation assays (Figure S1B, Supporting Information), and EdU incorporation assays (Figure S1C, Supporting Information). Under identical concentrations of lenvatinib, lenvatinib-resistant cells displayed markedly enhanced proliferative capacity and significantly reduced apoptosis compared to parental cells. To mimic the hypoxic conditions present in vivo, HCC cells were cultured in a hypoxic chamber under a strictly controlled gas environment (1% O₂, 5% CO₂, and 94% N₂) to establish hypoxic cell models. The successful induction of hypoxia was validated by immunoblotting analysis of hypoxia-inducible factor-1α (HIF-1α), which was markedly upregulated in hypoxic HCC cells compared to that in normoxic controls (Fig. 1C). We further evaluated the relationship between the hypoxic microenvironment and lenvatinib resistance in HCC. Notably, under hypoxic conditions, HCC cells exhibited significantly increased IC_50_ values for lenvatinib compared with those cultured under normoxic conditions (Fig. 1D).

Transcriptome sequencing was performed on both cell models to identify differentially expressed genes (DEGs) between lenvatinib-resistant and parental cells as well as between hypoxic and normoxic cells. DEGs were screened based on the criteria of *p* < 0.05 and |log₂ fold change (logFC)| > 1. Intersection analysis of the two DEG sets identified 11 overlapping genes (Fig. 1E), among which six genes exhibiting inconsistent expression patterns were excluded (Fig. 1F). Subsequently, the remaining five candidate genes (SERPINE2, ATF3, LPAR1, PIWIL2, and CAMK4) were further analyzed using data from the Liver Hepatocellular Carcinoma (LIHC) cohort in The Cancer Genome Atlas (TCGA) database (Figure S1D-F, Supporting Information). In parallel, reverse transcription quantitative PCR (RT–qPCR) was performed on 20 freshly collected lenvatinib-resistant HCC tumor samples and 20 lenvatinib-sensitive HCC tumor samples (Fig. 1G). The results indicated that the mRNA expression levels of ATF3 and PIWIL2 were not significantly different between the lenvatinib-resistant and lenvatinib-sensitive groups during clinical tissue validation (*p* > 0.05), leading to their exclusion. TCGA analysis revealed that the expression of LPAR1 and CAMK4 in normal tissues was significantly higher than that in tumor tissues. Although they displayed a slight trend during clinical tissue validation, these variation trends could not be reliably quantified and failed to demonstrate the prognostic significance associated with lenvatinib resistance, resulting in their exclusion. Only SERPINE2 was significantly upregulated in lenvatinib-resistant clinical samples and was correlated with poor clinical prognosis. Based on these results, serine protease inhibitor family E member 2 (SERPINE2) was ultimately identified as the key target gene. To further validate SERPINE2 expression, protein lysates were extracted from lenvatinib-resistant and parental cells, as well as from hypoxic and normoxic cells, followed by western blot analysis. The results demonstrated that SERPINE2 expression was significantly elevated in lenvatinib-resistant cells compared with that in parental cells (Fig. 1H) and in hypoxic cells compared with that in normoxic cells (Fig. 1I).

To further elucidate the relationship between hypoxia, lenvatinib resistance, and SERPINE2 upregulation, we treated parental cells with lenvatinib at the IC₅₀ concentration for 48 h. The results showed that SERPINE2 expression levels were significantly elevated, indicating that lenvatinib-induced cellular stress could serve as an independent trigger for SERPINE2 upregulation. Furthermore, we evaluated whether upregulation of SERPINE2 in lenvatinib-resistant cells relies on continuous drug stimulation. We conducted a washout experiment by culturing the resistant cells in lenvatinib-free medium for two weeks. Compared to parental cells, SERPINE2 expression remained constitutively high in resistant cells (Figure S1G, Supporting Information). These results suggest that chronic lenvatinib selection pressure induces stable phenotypic memory, enabling resistant cells to maintain robust SERPINE2 overexpression, independent of immediate hypoxic or pharmacological stimuli.

### SERPINE2 is upregulated in hepatocellular carcinoma and associated with poor prognosis, exhibiting oncogenic functions

**Fig. 2:**
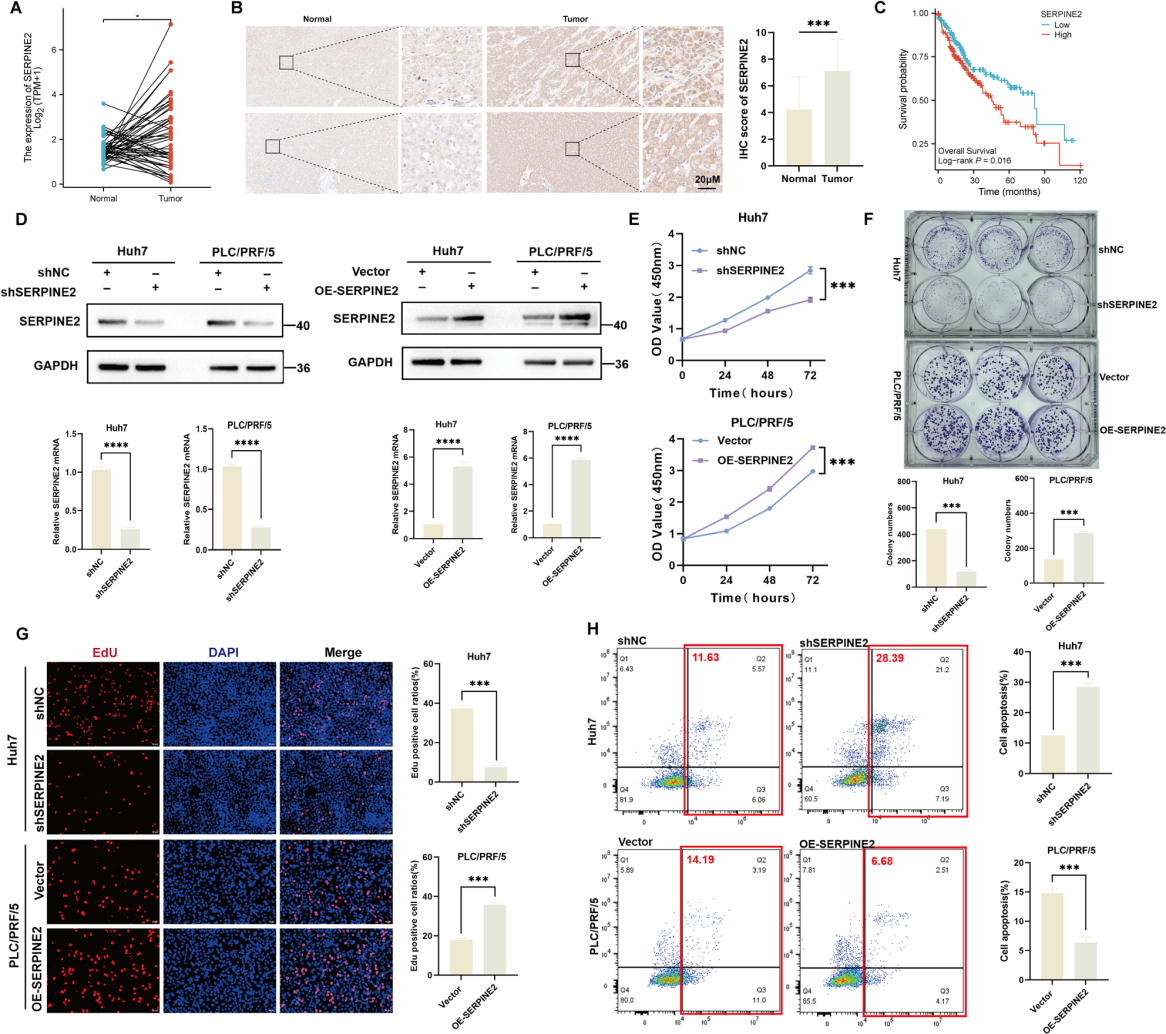
SERPINE2 is upregulated in hepatocellular carcinoma and functions as an oncogenic factor associated with poor prognosis. **A** Comparison of SERPINE2 mRNA expression levels between HCC tissues and paired adjacent non-tumorous liver tissues in the TCGA-LIHC cohort. **B** Representative immunohistochemical staining images and quantitative analysis of SERPINE2 protein expression in 18 paired HCC tumor tissues and adjacent non-tumorous liver tissues. **C** Kaplan–Meier survival analysis of overall survival in HCC patients from the TCGA cohort stratified according to SERPINE2 expression levels. **D** Validation of SERPINE2 knockdown and overexpression efficiencies at the mRNA and protein levels in Huh7 and PLC/PRF/5 cells by RT–qPCR and Western blotting, respectively; GAPDH served as an internal control. **E** CCK-8 assays showing the effects of SERPINE2 knockdown or overexpression on the proliferation of Huh7 and PLC/PRF/5 cells. **F** Colony formation assays demonstrating that SERPINE2 knockdown suppresses, whereas SERPINE2 overexpression enhances, the clonogenic capacity of HCC cells. **G** EdU incorporation assays assessing cell proliferation following SERPINE2 knockdown or overexpression in HCC cells. **H** Flow cytometric analysis of apoptosis in Huh7 and PLC/PRF/5 cells after SERPINE2 knockdown or overexpression. Data are presented as mean ± SD. Statistical analyses were performed using Student’s t-test or one-way ANOVA as appropriate. Statistical significance is indicated as ns, not significant; *, *p* < 0.05; **, *p* < 0.01; ***, *p* < 0.001; ****, *p*＜0.0001

To investigate the expression profile and potential role of SERPINE2 in HCC, we analyzed the publicly available TCGA-LIHC dataset. The results revealed that the mRNA expression levels of SERPINE2 were significantly higher in tumor tissues than in paired normal tissues. (Fig. 2A). To assess SERPINE2 expression at the protein level, IHC analysis was performed on 18 paired HCC tumor tissues and the corresponding adjacent non-tumorous liver tissues from our study cohort. The results demonstrated that SERPINE2 protein expression was significantly elevated in tumor tissues relative to that in normal liver tissues (Fig. 2B). In addition, survival analysis of the TCGA cohort showed that high SERPINE2 expression was significantly associated with shorter overall survival (OS) in patients (Fig. 2C). Collectively, these findings indicate that SERPINE2 is aberrantly overexpressed in hepatocellular carcinoma and may serve as a potential prognostic biomarker for unfavorable clinical outcomes.

To investigate the oncogenic role of SERPINE2 in HCC, loss- and gain-of-function experiments were performed using Huh7 and PLC/PRF/5 cells. The efficiency of SERPINE2 knockdown and overexpression was validated at both the mRNA and protein levels compared to that of the negative controls (Fig. 2D). Next, we examined the role of SERPINE2 in the regulation of HCC cell growth. Knockdown of SERPINE2 in Huh7 and PLC/PRF/5 cells significantly suppressed cell proliferation capacity (Fig. 2E and Figure S2A, Supporting Information) and colony formation ability (Fig. 2F and Figure S2B, Supporting Information), and markedly reduced DNA synthesis activity, as assessed by EdU incorporation assays (Fig. 2G and Figure S2C, Supporting Information). In addition, flow cytometric analysis revealed that SERPINE2 knockdown significantly increased the apoptotic rate of HCC cells (Fig. 2H and Figure S2D, Supporting Information). Conversely, overexpression of SERPINE2 in Huh7 and PLC/PRF/5 cells significantly enhanced cell proliferation (Fig. 2E and Figure S2A, Supporting Information) and colony formation capacity(Fig. 2F and Figure S2B, Supporting Information), increased EdU incorporation (Fig. 2G and Figure S2C, Supporting Information), and markedly reduced apoptosis (Fig. 2H and Figure S2D, Supporting Information). Collectively, these results demonstrate that SERPINE2 plays a critical role in promoting HCC cell growth and survival, supporting its function as an oncogenic factor in hepatocellular carcinoma.

### SERPINE2 promotes lenvatinib resistance and is associated with poor therapeutic outcomes in hepatocellular carcinoma

**Fig. 3:**
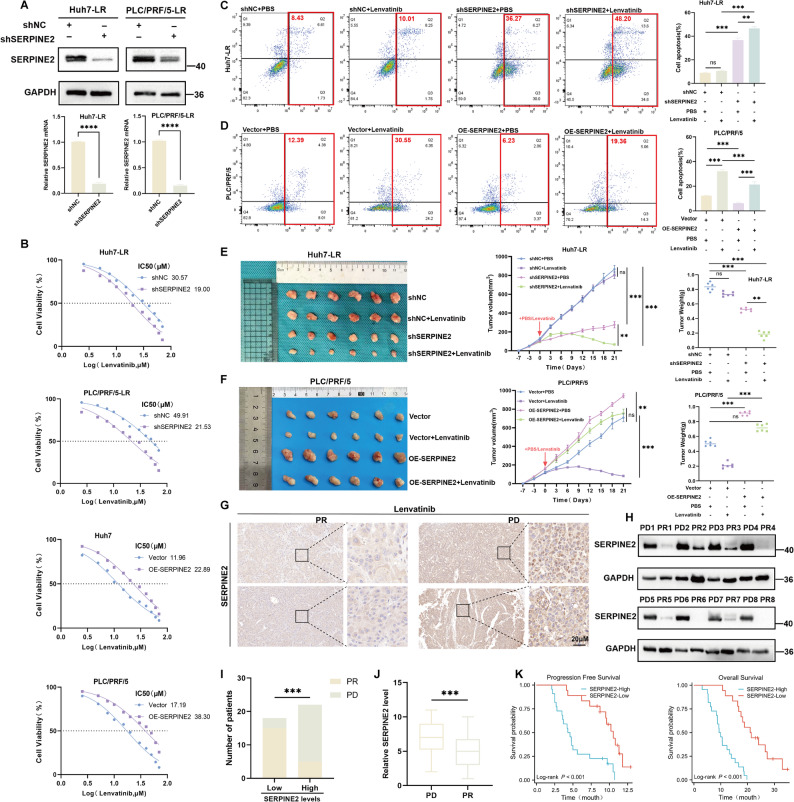
SERPINE2 promotes lenvatinib resistance in hepatocellular carcinoma in vitro and in vivo and is associated with poor clinical response. **A** RT–qPCR and Western blot analyses confirming the efficiency of SERPINE2 knockdown in Huh7-LR and PLC/PRF/5-LR cells and SERPINE2 overexpression in parental Huh7 and PLC/PRF/5 cells, compared with corresponding negative controls. **B** Determination of lenvatinib IC₅₀ values showing that SERPINE2 knockdown significantly decreased IC₅₀ in Huh7-LR and PLC/PRF/5-LR cells, whereas SERPINE2 overexpression markedly increased IC₅₀ in parental Huh7 and PLC/PRF/5 cells. **C** Flow cytometric analysis of apoptosis in lenvatinib-treated Huh7-LR cells following SERPINE2 knockdown, demonstrating enhanced drug sensitivity and increased apoptotic rates. **D** Flow cytometric analysis showing reduced apoptosis and enhanced lenvatinib resistance in PLC/PRF/5 cells overexpressing SERPINE2 after lenvatinib treatment. **E** Subcutaneous xenograft tumor growth of Huh7-LR cells with stable SERPINE2 knockdown treated with lenvatinib, showing significantly reduced tumor volume and weight. **F** Subcutaneous xenograft tumor growth of PLC/PRF/5 cells with stable SERPINE2 overexpression treated with lenvatinib, indicating enhanced tumor growth and reduced therapeutic efficacy of lenvatinib. **G** Representative immunohistochemical (IHC) staining of SERPINE2 in tumor tissues from lenvatinib-resistant (PD) and lenvatinib-sensitive (PR) HCC patients. **H** Western blot analysis of SERPINE2 expression in fresh tumor tissues from PD and PR patients. **I** Distribution of therapeutic response (PR vs. PD) according to SERPINE2 expression levels determined by IHC, showing a significantly higher proportion of PR cases in the SERPINE2-low group. **J** Box plot illustrating the association between SERPINE2 expression status and clinical response to lenvatinib. **K** Kaplan–Meier analyses of progression-free survival (PFS) and overall survival (OS) stratified by SERPINE2 expression levels, indicating poorer prognosis in patients with high SERPINE2 expression. Data are presented as mean ± SD. Statistical analyses were performed using Student’s t-test, one-way ANOVA, or log-rank test, as appropriate. Statistical significance is indicated as ns, not significant; *, *p* < 0.05; **, *p* < 0.01; ***, *p* < 0.001

Next, we investigated the role of SERPINE2 in promoting lenvatinib resistance in HCC. SERPINE2 knockdown was performed in Huh7-LR and PLC/PRF/5-LR cells, whereas SERPINE2 overexpression was conducted in parental Huh7 and PLC/PRF/5 cells. Compared to the negative controls, the efficiency of SERPINE2 silencing was validated at both the mRNA and protein levels (Fig. 3A). Functional assays demonstrated that knockdown of SERPINE2 significantly reduced the IC_50_ of lenvatinib in Huh7-LR and PLC/PRF/5-LR cells, whereas overexpression of SERPINE2 in parental Huh7 and PLC/PRF/5 cells markedly increased the IC_50_ of lenvatinib compared with the control groups (Fig. 3B). Flow cytometric analysis of apoptosis following lenvatinib treatment further revealed that SERPINE2 knockdown significantly enhanced lenvatinib sensitivity in Huh7-LR (Fig. 3C) and PLC/PRF/5-LR cells (Figure S3A, Supporting Information), as evidenced by the marked increase in apoptotic rates. In contrast, overexpression of SERPINE2 significantly enhanced lenvatinib resistance in Huh7 (Figure S3B, Supporting Information) and PLC/PRF/5 cells (Fig. 3D), accompanied by a significant reduction in apoptosis. To further validate the in vivo role of SERPINE2 in lenvatinib resistance, subcutaneous xenograft models were established using stable SERPINE2-knockdown Huh7-LR and stable SERPINE2-overexpressing PLC/PRF/5 cells. The results showed that SERPINE2 knockdown markedly enhanced tumor sensitivity to lenvatinib, leading to significantly reduced tumor volume and weight (Fig. 3E). IHC analysis of the tumor tissues revealed a significant decrease in the proliferation marker Ki-67 (Figure S3C, Supporting Information). Conversely, SERPINE2 overexpression enhanced lenvatinib resistance in vivo, attenuated the antitumor efficacy of lenvatinib (Fig. 3F), and resulted in elevated Ki-67 expression in tumor tissues (Figure S3D, Supporting Information). Collectively, these findings demonstrate that SERPINE2 plays a critical role in mediating lenvatinib resistance in hepatocellular, both in vitro and in vivo.

To further investigate the clinical relevance of SERPINE2 in lenvatinib resistance and prognosis of HCC, IHC analysis was performed on tumor tissues from 20 lenvatinib-resistant HCC patients with progressive disease (PD) and 20 lenvatinib-sensitive HCC patients with partial response (PR) from our study cohort (Fig. 3G). In addition, western blot analysis was conducted on tumor tissues from eight freshly collected PD patients and eight PR patients (Fig. 3H). The results demonstrated that SERPINE2 expression levels were significantly higher in tumor tissues from lenvatinib-resistant patients with HCC than in those from lenvatinib-sensitive patients. Based on IHC staining intensity, patients were stratified into SERPINE2 high- and low-expression groups. Among the 18 patients in the SERPINE2 low-expression group, 15 patients (83.3%) achieved a partial response, which was significantly higher than that observed in the SERPINE2 high-expression group, in which only 5 of 22 patients (22.7%) achieved a partial response (*p* < 0.05) (Fig. 3I). Notably, the majority of PR cases were distributed in the SERPINE2 low expression group, whereas most PD cases were observed in the SERPINE2 high expression group (Fig. 3J). Furthermore, high SERPINE2 expression was significantly associated with shorter progression-free survival (PFS) and OS in patients with HCC receiving lenvatinib treatment (Fig. 3K). Collectively, these findings indicate that elevated SERPINE2 expression is clinically associated with unfavorable therapeutic outcomes and poor prognosis in patients with lenvatinib-treated hepatocellular carcinoma patients.

### SERPINE2 promotes GCLC transcription to suppress ferroptosis and induce lenvatinib resistance in hepatocellular carcinoma

**Fig. 4:**
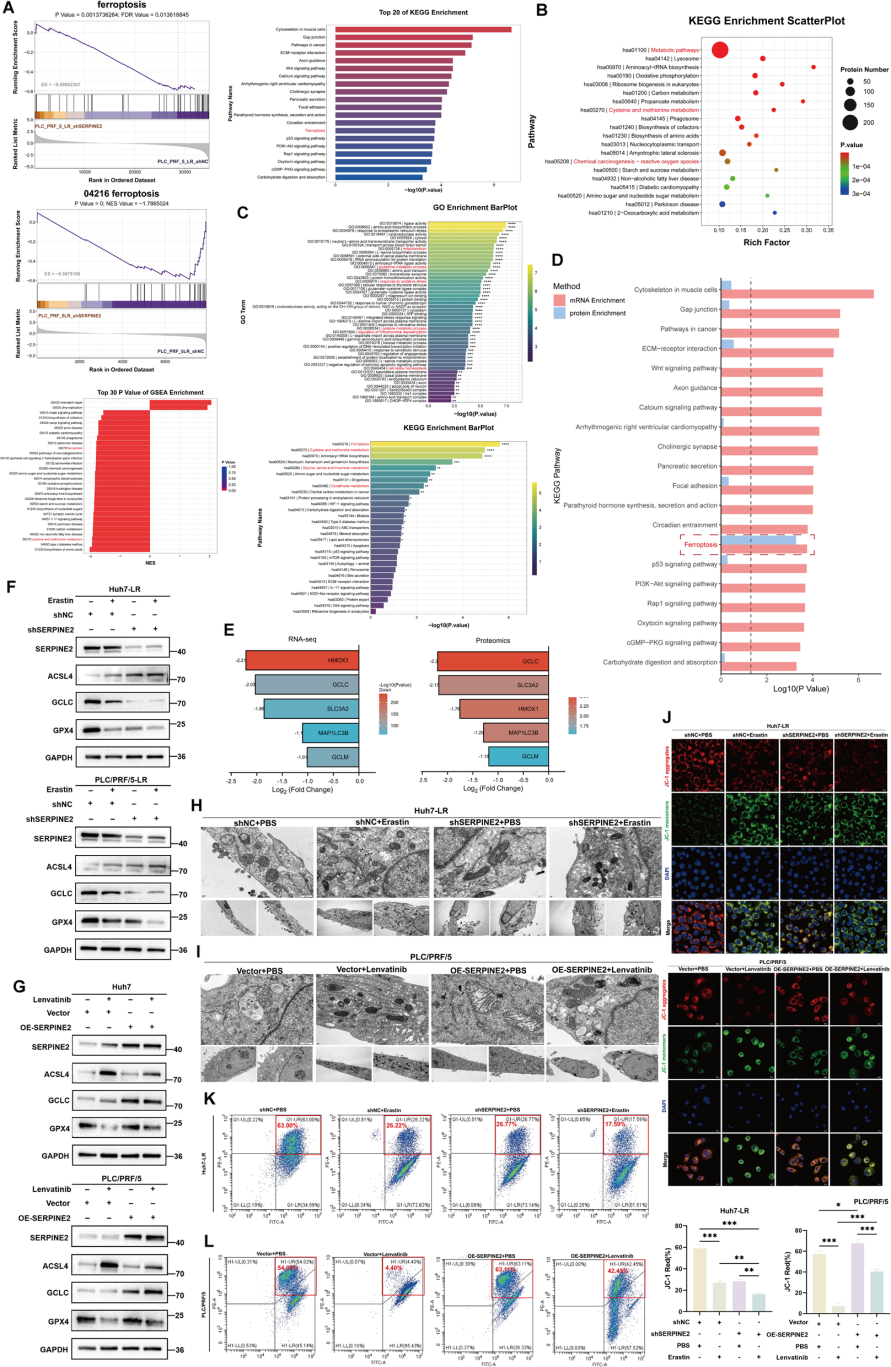
SERPINE2 suppresses ferroptosis in lenvatinib-resistant hepatocellular carcinoma by promoting GCLC transcription and maintaining redox homeostasis and mitochondrial function. **A** Gene set enrichment analysis (GSEA) of transcriptomic data from SERPINE2-knockdown PLC/PRF/5-LR cells versus control cells, showing significant enrichment of ferroptosis-related gene sets upon SERPINE2 depletion. **B** KEGG pathway analysis of proteomic data from SERPINE2-knockdown PLC/PRF/5-LR cells, demonstrating significant enrichment of ferroptosis-associated pathways, including metabolic pathways, cysteine and methionine metabolism, oxidative phosphorylation, and reactive oxygen species–related processes. **C** Integrated multi-omics analysis of transcriptomic and proteomic datasets from stable SERPINE2-knockdown PLC/PRF/5-LR cells and control cells. KEGG and GO enrichment analyses of commonly downregulated genes/proteins revealed significant enrichment in ferroptosis-related pathways, including glutathione metabolism, cysteine and methionine metabolism, and redox homeostasis. **D** Combined KEGG analysis of transcriptomic and proteomic data showing that the ferroptosis pathway was the most significantly enriched among the differential pathways. **E** Heatmap and ranking of ferroptosis-related differentially expressed genes/proteins identified by integrated transcriptomic and proteomic analyses, highlighting GCLC as the most prominently altered candidate. **F** Western blot analysis of ferroptosis marker proteins in SERPINE2-knockdown lenvatinib-resistant cells, showing decreased GPX4 expression and increased ACSL4 expression, consistent with the effects of the ferroptosis inducer erastin. **G** Western blot analysis demonstrating that SERPINE2 overexpression in parental cells increased GPX4 expression and decreased ACSL4 expression, thereby counteracting lenvatinib-induced ferroptosis. **H, I** Representative transmission electron microscopy images of mitochondrial ultrastructure in hepatocellular carcinoma cells with different SERPINE2 expression statuses following erastin or lenvatinib treatment. SERPINE2 knockdown or erastin treatment induced typical ferroptosis-associated mitochondrial abnormalities, whereas SERPINE2 overexpression alleviated lenvatinib-induced mitochondrial damage. **J**–**L** Assessment of mitochondrial membrane potential (ΔΨm) by JC-1 staining and flow cytometry. SERPINE2 knockdown significantly reduced ΔΨm in Huh7-LR and PLC/PRF/5-LR cells, with effects comparable to and synergistic with erastin treatment; conversely, SERPINE2 overexpression increased ΔΨm in parental cells and mitigated lenvatinib-induced mitochondrial dysfunction. Data are presented as mean ± SD. Statistical significance was determined using Student’s t-test or one-way ANOVA, as appropriate. Statistical notations: ns, not significant; *, *p* < 0.05; **, *p* < 0.01; ***, *p* < 0.001

To elucidate the molecular mechanisms by which SERPINE2 promotes lenvatinib resistance in HCC, RNA-seq and proteomic analyses were performed on stable SERPINE2-knockdown PLC/PRF/5-LR cells and the corresponding control cells, followed by integrated multi-omics analysis. KEGG pathway analysis of differentially expressed genes identified by RNA-seq revealed a significant enrichment in ferroptosis-related pathways. Consistently, gene set enrichment analysis (GSEA) demonstrated a strong association between SERPINE2 knockdown and ferroptosis-related gene signatures (Fig. 4A). KEGG pathway analysis of differentially expressed proteins from proteomic profiling revealed prominent enrichment in ferroptosis-associated pathways, including metabolic pathways, cysteine and methionine metabolism, oxidative phosphorylation, and reactive oxygen species–related processes. GSEA of the proteomic data showed that SERPINE2 knockdown was significantly correlated with ferroptosis-related pathways (Fig. 4B). Subsequent integrative multi-omics analyses of transcriptomic and proteomic datasets were performed. KEGG enrichment analysis of the commonly downregulated genes and proteins revealed significant enrichment in ferroptosis and its associated pathways, including cysteine, methionine, and glutathione metabolism. Gene Ontology (GO) enrichment analysis further indicated strong associations with ferroptosis-related biological processes, such as cellular redox homeostasis and cysteine metabolism (Fig. 4C). Collectively, these findings suggest that SERPINE2 promotes lenvatinib resistance in HCC by modulating redox homeostasis and suppressing ferroptosis (Fig. 4D).

Based on integrative multi-omics analysis, ferroptosis-related differentially expressed genes and proteins were further screened, among which GCLC exhibited the most pronounced alteration (Fig. 4E). First, we examined the regulatory effect of SERPINE2 on GCLC expression. Western blotting and RT–qPCR analyses demonstrated that knockdown of SERPINE2 in lenvatinib-resistant cell lines significantly reduced GCLC protein and mRNA levels, whereas overexpression of SERPINE2 in parental cells markedly increased GCLC expression at both protein (Figure S4A, Supporting Information) and mRNA (Figure S4B, Supporting Information) levels. Erastin, a well-established inhibitor of the cystine/glutamate antiporter system Xc⁻, is known to induce ferroptosis [22]. We used erastin to further assess whether suppression of SERPINE2 enhances cellular susceptibility to ferroptosis. First, we investigated the effects of SERPINE2 on ferroptosis-related marker proteins. Glutathione peroxidase 4 (GPX4) plays a critical role in suppressing ferroptosis by detoxifying phospholipid hydroperoxides, whereas acyl-CoA synthetase long-chain family member 4 (ACSL4) promotes ferroptosis by facilitating esterification of polyunsaturated fatty acids into phospholipids, thereby enhancing lipid peroxidation [23]. Accordingly, GPX4 and ACSL4 expression levels were examined following SERPINE2 knockdown or overexpression, respectively. Western blot analysis revealed that SERPINE2 knockdown in lenvatinib-resistant cells resulted in a significant decrease in GPX4 protein levels and a concomitant increase in ACSL4 expression, a pattern consistent with the effects induced by the ferroptosis inducer erastin (Fig. 4F). In contrast, overexpression of SERPINE2 in parental cells significantly increased GPX4 protein levels, while reducing ACSL4 expression, an effect that counteracted the action of lenvatinib (Fig. 4G). Ferroptosis is frequently accompanied by mitochondrial dysfunction, which is characterized by structural abnormalities and loss of mitochondrial membrane potential [24]. We examined mitochondrial morphology in HCC cells treated with erastin (10µM) using transmission electron microscopy (TEM). The results showed that erastin-induced ferroptosis is characterized by mitochondrial shrinkage, membrane rupture, distorted cristae with reduced density, and partial or complete loss of cristae. These morphological changes were consistent with those observed following SERPINE2 knockdown in lenvatinib-resistant cells, and were further exacerbated when SERPINE2 knockdown cells were treated with erastin. Similarly, treatment of parental cells with lenvatinib induced mitochondrial alterations comparable to those caused by erastin, whereas SERPINE2 overexpression alleviated lenvatinib-induced mitochondrial damage (Fig. 4H–I and Figure S4C-D, Supporting Information). Mitochondrial membrane potential (ΔΨm) was assessed using the JC-1 assay to evaluate mitochondrial function. In Huh7-LR and PLC/PRF/5-LR cells, SERPINE2 knockdown resulted in a significant reduction in mitochondrial membrane potential compared to control cells, an effect comparable to that induced by erastin and synergistic with erastin treatment. Conversely, overexpression of SERPINE2 in Huh7 and PLC/PRF/5 cells significantly increased the mitochondrial membrane potential relative to control cells and mitigated the effects of lenvatinib (Fig. 4J–L and Figure S4E-G, Supporting Information).

**Fig. 5:**
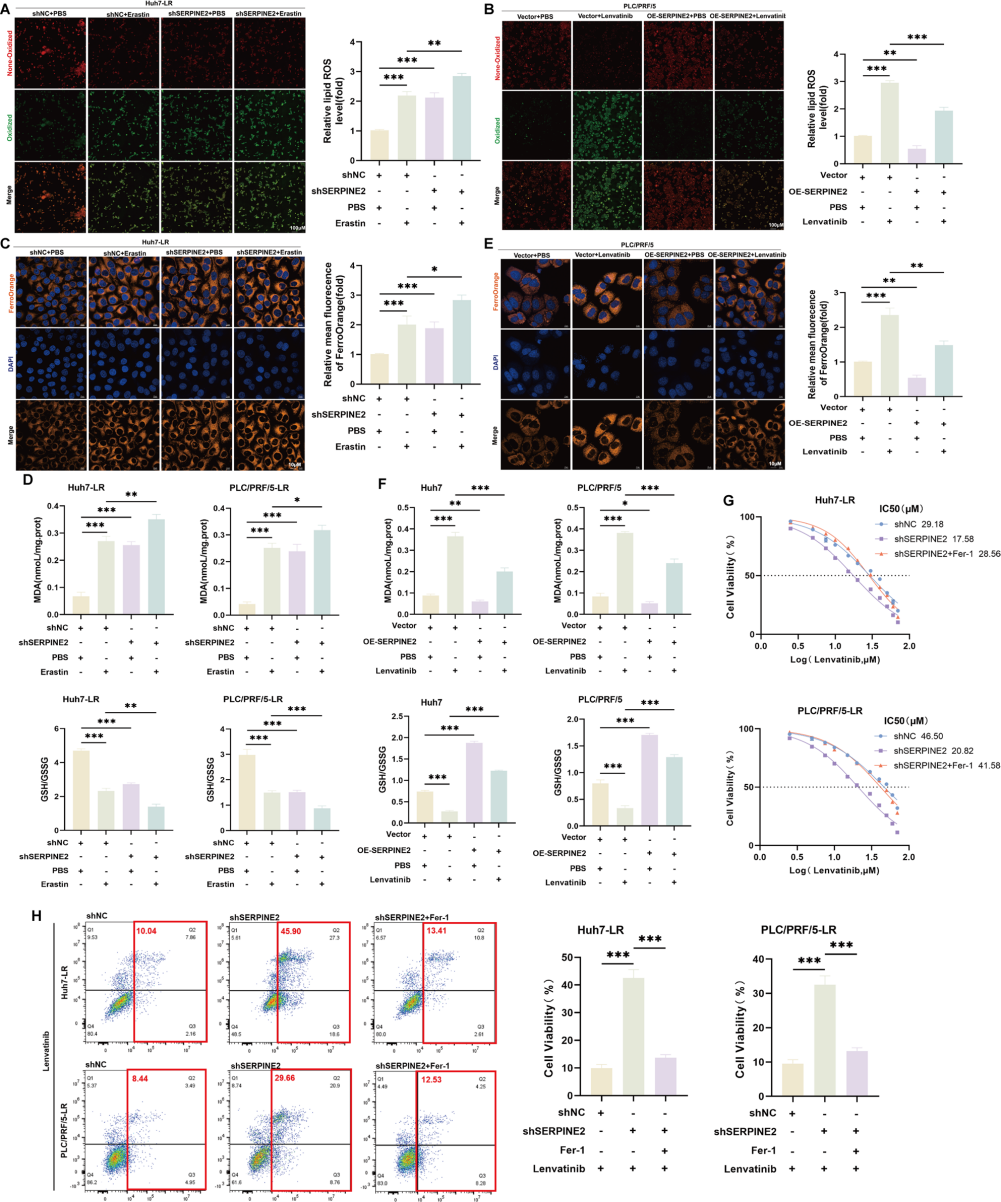
SERPINE2 promotes lenvatinib resistance in hepatocellular carcinoma by inhibiting lipid peroxidation–induced ferroptosis. **A** Intracellular lipid reactive oxygen species (ROS) levels in SERPINE2-silenced Huh7-LR cells detected by C11-BODIPY staining, showing a marked increase in lipid ROS accumulation, comparable to and synergistic with erastin treatment. **B** Lipid ROS levels in parental PLC/PRF/5 cells treated with lenvatinib, with or without SERPINE2 overexpression. SERPINE2 overexpression markedly attenuated lenvatinib-induced lipid ROS accumulation. **C** Intracellular ferrous iron (Fe²⁺) levels in SERPINE2-knockdown Huh7-LR cells, showing a significant increase consistent with ferroptotic induction. **D** Quantification of malondialdehyde (MDA) levels and the GSH/GSSG ratio in SERPINE2-silenced lenvatinib-resistant cells, indicating enhanced lipid peroxidation and disrupted redox balance, similar to the effects of erastin treatment. **E** Intracellular Fe²⁺ levels in parental PLC/PRF/5 cells overexpressing SERPINE2 following lenvatinib treatment, demonstrating reduced iron accumulation. **F** MDA levels and GSH/GSSG ratios in SERPINE2-overexpressing parental cells, indicating enhanced antioxidant capacity and alleviation of lenvatinib-induced oxidative stress. **G** IC₅₀ values of lenvatinib in SERPINE2-knockdown Huh7-LR and PLC/PRF/5-LR cells treated with or without the ferroptosis inhibitor Ferrostatin-1 (Fer-1). Fer-1 significantly reversed the increased lenvatinib sensitivity caused by SERPINE2 depletion. **H** Flow cytometric analysis of apoptosis in SERPINE2-silenced lenvatinib-resistant cells treated with lenvatinib, with or without Fer-1, showing that Fer-1 partially rescued apoptosis induced by SERPINE2 knockdown. Data are presented as mean ± SD. Statistical analyses were performed using Student’s t-test or one-way ANOVA as appropriate. Statistical significance is indicated as follows: ns, not significant; *, *p* < 0.05; **, *p* < 0.01; ***, *p* < 0.001

Next, we investigated the effect of SERPINE2 on cellular redox homeostasis. Lipid peroxidation is a hallmark of ferroptosis that directly damages cellular membranes and ultimately triggers ferroptotic cell death [25]. To assess lipid peroxidation, intracellular lipid reactive oxygen species (ROS) accumulation was measured using the lipid peroxidation–sensitive fluorescent probe C11-BODIPY. In Huh7-LR and PLC/PRF/5-LR cells, knockdown of SERPINE2 significantly increased intracellular lipid ROS levels compared with control cells. This effect was comparable to that induced by the ferroptosis inducer erastin, and was further enhanced when SERPINE2 knockdown was combined with erastin treatment (Fig. 5A and Figure S5A, Supporting Information). In contrast, treatment of parental Huh7 and PLC/PRF/5 cells with lenvatinib markedly increased lipid ROS accumulation, whereas overexpression of SERPINE2 significantly reduced lipid ROS levels, thereby attenuating the effects of lenvatinib (Fig. 5B and Figure S5B, Supporting Information). Ferroptosis is driven by redox imbalance, and malondialdehyde (MDA) levels, glutathione/oxidized glutathione (GSH/GSSG) ratio, and intracellular ferrous iron (Fe²⁺) levels are widely recognized biomarkers of ferroptosis. Compared to control cells, SERPINE2 knockdown resulted in a significant increase in intracellular Fe²⁺ levels (Fig. 5C and Figure S5C, Supporting Information) and MDA levels, accompanied by a marked decrease in the GSH/GSSG ratio (Fig. 5D), consistent with the effects observed following erastin treatment. Conversely, overexpression of SERPINE2 significantly reduced intracellular Fe²⁺ levels (Fig. 5E and Figure S5D, Supporting Information) and MDA levels and markedly increased the GSH/GSSG ratio (Fig. 5F), thereby mitigating the effects of lenvatinib.

We further investigated whether the SERPINE2-mediated regulation of ferroptosis contributes to lenvatinib resistance in HCC. Following SERPINE2 knockdown in Huh7-LR and PLC/PRF/5-LR cells, the ferroptosis inhibitor ferrostatin-1 (Fer-1) was applied [26]. The results showed that, compared with control cells, SERPINE2 knockdown significantly reduced the IC_50_ of lenvatinib in lenvatinib-resistant HCC cells, whereas this effect was markedly reversed by Fer-1 treatment (Fig. 5G). Flow cytometric analysis of apoptosis further demonstrated that under lenvatinib treatment, SERPINE2 knockdown significantly increased apoptotic rates in lenvatinib-resistant HCC cells, while Fer-1 treatment effectively attenuated this increase in apoptosis (Fig. 5H). Collectively, these results indicate that SERPINE2 suppresses ferroptosis by promoting GCLC transcription, thereby contributing to lenvatinib resistance in hepatocellular carcinoma.

### SERPINE2 activates NRF2 nuclear translocation to induce GCLC transcription

**Fig. 6:**
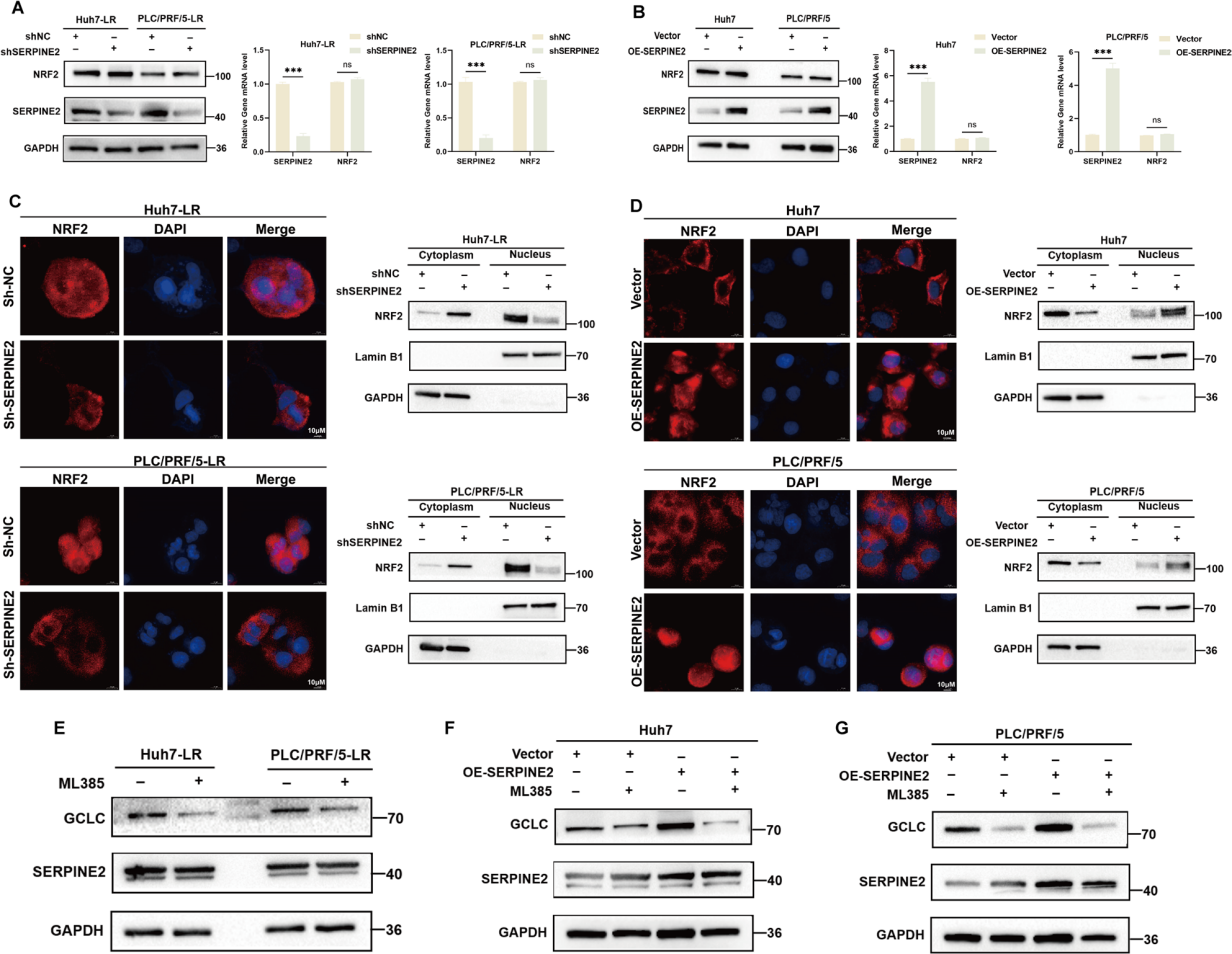
SERPINE2 upregulates GCLC transcription by promoting NRF2 nuclear translocation. **A** In lenvatinib-resistant hepatocellular carcinoma cells, knockdown of SERPINE2 did not alter NRF2 protein or mRNA levels, as determined by Western blotting and RT–qPCR. **B** Similarly, overexpression of SERPINE2 in parental Huh7 and PLC/PRF/5 cells did not significantly affect NRF2 protein or mRNA expression. **C** Nuclear–cytoplasmic fractionation combined with Western blotting and immunofluorescence analysis showed that SERPINE2 knockdown resulted in cytoplasmic retention of NRF2 and a marked reduction of its nuclear localization. **D** In contrast, SERPINE2 overexpression in parental cells significantly promoted NRF2 nuclear translocation and increased its nuclear accumulation. **E** Treatment of lenvatinib-resistant cells with the NRF2-specific inhibitor ML385 markedly reduced GCLC protein expression, as shown by Western blot analysis. **F**, **G** In parental cells overexpressing SERPINE2, combined treatment with ML385 significantly decreased GCLC protein and mRNA levels, indicating that NRF2 inhibition effectively blocks SERPINE2-mediated upregulation of GCLC. Data are presented as mean ± SD. Statistical analyses were performed using Student’s t-test or one-way ANOVA, as appropriate. Statistical significance is indicated as follows: ns, not significant; *, *p* < 0.05; **, *p* < 0.01; ***, *p* < 0.001

As a member of the serine protease inhibitor family, SERPINE2 is predominantly localized in the cytoplasm and is therefore unlikely to directly regulate the transcription of GCLC. Given that nuclear factor erythroid 2–related factor 2 (NRF2) is a well-established master regulator of ferroptosis and antioxidant responses, and that GCLC is a canonical transcriptional target of NRF2 [16], we next investigated whether SERPINE2 regulates GCLC expression through modulation of NRF2. SERPINE2 was silenced in lenvatinib-resistant cell lines and overexpressed in parental cells. NRF2 protein and mRNA levels were subsequently examined using western blotting and RT–qPCR. The results showed that Neither knockdown (Fig. 6A) nor overexpression (Fig. 6B) of SERPINE2 significantly affected NRF2 protein or mRNA expression levels, indicating that SERPINE2 does not regulate GCLC transcription by altering NRF2 expression. Next, we examined the effect of SERPINE2 on NRF2 nuclear translocation. Western blotting of the cytoplasmic and nuclear fractions, together with immunofluorescence analysis, demonstrated that SERPINE2 knockdown resulted in a marked accumulation of NRF2 in the cytoplasm and a concomitant reduction in NRF2 levels in the nucleus (Fig. 6C). In contrast, SERPINE2 overexpression significantly enhanced the nuclear localization of NRF2 (Fig. 6D). ML385 is a selective NRF2 inhibitor that directly binds to the DNA-binding domain of NRF2, thereby preventing its interaction with antioxidant response elements (AREs) and suppressing the transcription of NRF2 target genes [27]. Treatment of lenvatinib-resistant cells with ML385 resulted in a significant reduction in GCLC expression, as determined by western blotting (Fig. 6E). Furthermore, ML385 treatment markedly attenuated SERPINE2 overexpression–induced upregulation of GCLC in parental cells (Fig. 6F–G), indicating that inhibition of NRF2 effectively blocked the regulatory effect of SERPINE2 on GCLC expression. Collectively, these findings demonstrated that SERPINE2 regulates GCLC transcription by promoting NRF2 nuclear translocation rather than by altering NRF2 expression levels.

### SERPINE2 activates the JAK2/STAT3 signaling pathway, and STAT3 interacts with NRF2 to promote its nuclear translocation

**Fig. 7:**
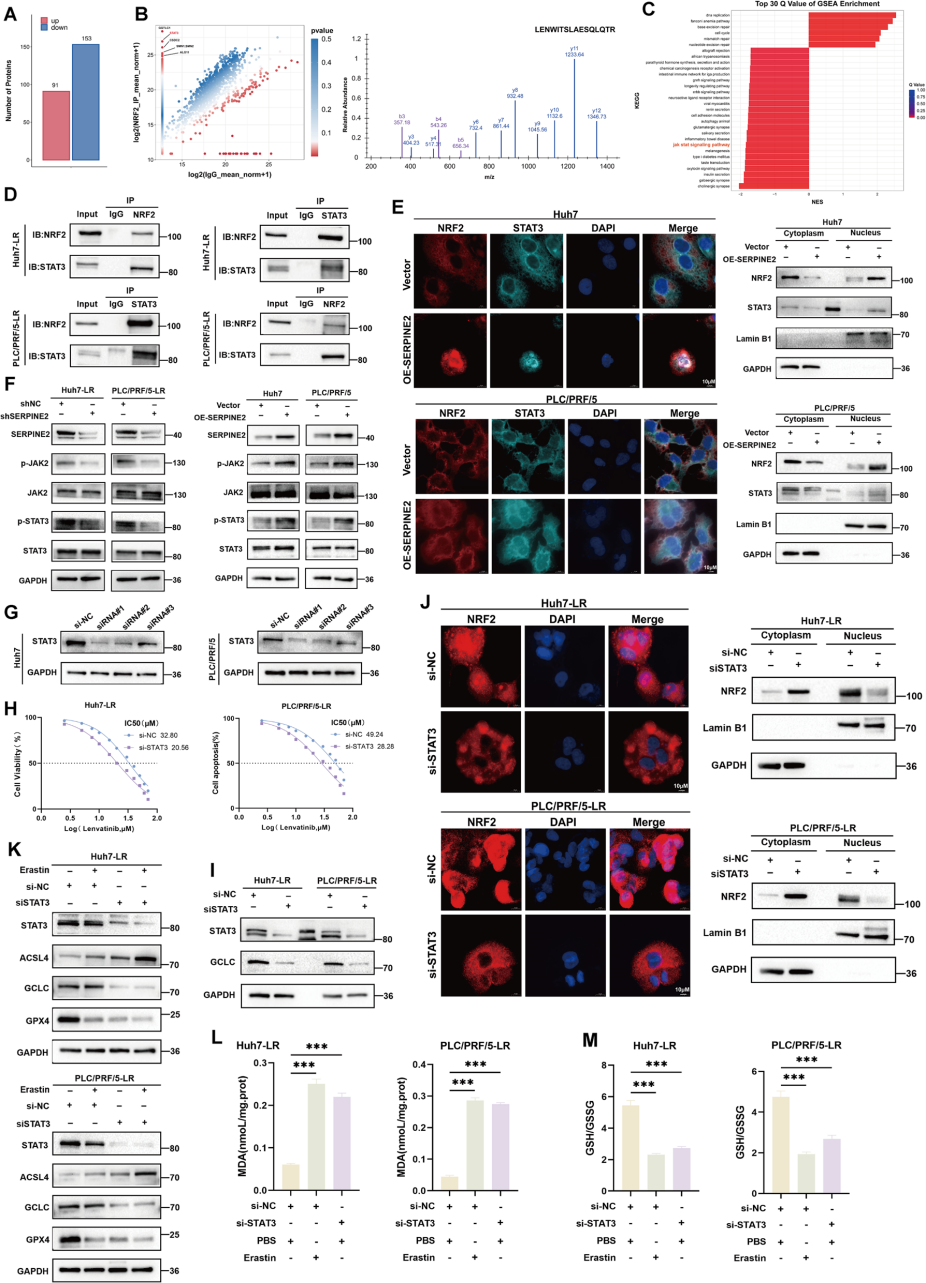
SERPINE2 promotes STAT3–NRF2 interaction and drives NRF2 nuclear translocation by activating the JAK2/STAT3 signaling pathway. **A** Quantitative proteomic analysis in PLC/PRF/5-LR cells identifying NRF2-interacting proteins, revealing 91 significantly upregulated and 153 significantly downregulated interacting proteins. **B** Volcano plot showing the top ten significantly upregulated NRF2-interacting proteins, among which STAT3 was identified; representative secondary mass spectrometry spectra confirming the interaction between STAT3 and NRF2 are shown. **C** Gene set enrichment analysis (GSEA) based on transcriptomic profiling of SERPINE2-stably silenced PLC/PRF/5-LR cells, demonstrating significant suppression of the JAK/STAT signaling pathway. **D** Co-immunoprecipitation (Co-IP) assays showing that endogenous NRF2 interacts with endogenous STAT3 in lenvatinib-resistant HCC cells. **E** Western blot and immunofluorescence analyses showing that SERPINE2 overexpression in parental HCC cells reduces cytoplasmic levels of NRF2 and STAT3 while markedly increasing their nuclear accumulation, with evident co-localization in both the cytoplasm and nucleus. **F** Western blot analysis showing that SERPINE2 knockdown in lenvatinib-resistant cells significantly decreases p-JAK2 and p-STAT3 levels, whereas SERPINE2 overexpression in parental cells markedly increases p-JAK2 and p-STAT3 levels, indicating activation of the JAK2/STAT3 pathway. **G** Validation of STAT3 knockdown efficiency using siRNAs, with siRNA#1 selected for subsequent experiments due to its highest silencing efficiency. **H** CCK-8 assays showing that STAT3 knockdown significantly reduces the IC₅₀ of lenvatinib in Huh7-LR and PLC/PRF/5-LR cells. **I** Western blot analysis showing that GCLC protein expression is markedly reduced in lenvatinib-resistant cells following STAT3 knockdown. **J** Nuclear/cytoplasmic fractionation combined with Western blot and immunofluorescence analyses showing that STAT3 knockdown causes NRF2 to be retained predominantly in the cytoplasm, with a significant reduction in nuclear NRF2 levels. **K** Western blot analysis of ferroptosis-related proteins showing that STAT3 knockdown increases ACSL4 expression and decreases GPX4 expression in lenvatinib-resistant cells. **L** Quantification of malondialdehyde (MDA) levels showing a significant increase in lenvatinib-resistant cells after STAT3 knockdown. **M** Analysis of the GSH/GSSG ratio showing a significant decrease following STAT3 knockdown in lenvatinib-resistant cells. Data are presented as mean ± standard deviation (SD). Statistical analyses were performed using Student’s t test or one-way analysis of variance (ANOVA). Statistical significance is indicated as follows: ns, not significant; *, *p* < 0.05; **, *p* < 0.01; ***, *p* < 0.001

Next, we sought to elucidate the molecular mechanism by which SERPINE2 promotes NRF2 nuclear translocation. Quantitative mass spectrometry–based proteomic analysis was performed in PLC/PRF/5-LR cells to identify the NRF2-interacting proteins. A total of 91 proteins exhibiting significantly increased binding and 153 proteins exhibiting significantly decreased binding to NRF2 were identified (Fig. 7A). Among the top ten upregulated interacting proteins, signal transducer and activator of transcription 3 (STAT3) was identified as a prominent candidate (Fig. 7B). Consistent with these findings, transcriptomic analysis of PLC/PRF/5-LR cells following SERPINE2 knockdown revealed significant suppression of the JAK/STAT signaling pathway, as demonstrated by GSEA (Fig. 7C). Given that STAT3 is a well-established transcription factor, we hypothesized that SERPINE2 activates the JAK2/STAT3 signaling pathway, and that activated STAT3 interacts with NRF2 to facilitate its nuclear translocation. To test this hypothesis, co-immunoprecipitation (Co-IP) assays were performed, which demonstrated that endogenous NRF2 physically interacted with endogenous STAT3 in lenvatinib-resistant HCC cells (Fig. 7D). Furthermore, overexpression of SERPINE2 in parental HCC cells resulted in a marked decrease in cytoplasmic NRF2 and STAT3 protein levels, accompanied by a significant increase in their nuclear localization, as determined by western blot and immunofluorescence analyses. Notably, NRF2 and STAT3 were co-localized in both the cytoplasm and nucleus (Fig. 7E). To further delineate the precise interaction domains between STAT3 and NRF2, we constructed a series of truncation mutants of both STAT3 and NRF2 (Figure S6A, Supporting Information). Subsequent Co-IP mapping assays revealed that the amino acid region 322–495 of STAT3 (Figure S6B, Supporting Information) and the amino acid region 228–605 of NRF2 (Figure S6C, Supporting Information) constitute their specific physical binding interface. This result established a specific structural basis for the interaction between STAT3 and NRF2. We further examined the effect of SERPINE2 on JAK2/STAT3 signaling activation. SERPINE2 was silenced in lenvatinib-resistant cells and overexpressed in parental cells, followed by western blot analysis. SERPINE2 knockdown significantly reduced the levels of phosphorylated JAK2 (p-JAK2) and phosphorylated STAT3 (p-STAT3), indicating suppression of the JAK2/STAT3 signaling pathway. Conversely, overexpression of SERPINE2 markedly increased p-JAK2 and p-STAT3 levels, indicating activation of this pathway (Fig. 7F).

To further validate the role of STAT3 in SERPINE2-mediated lenvatinib resistance, STAT3 expression was silenced using small interfering RNA (siRNA). Among the tested siRNAs, siRNA#1 achieved the most efficient knockdown, and was therefore selected for subsequent experiments (Fig. 7G). STAT3 knockdown significantly reduced the IC_50_ of lenvatinib in both Huh7-LR and PLC/PRF/5-LR cells compared to that in the control cells (Fig. 7H). Western blot analysis further demonstrated that GCLC expression was markedly decreased following STAT3 silencing (Fig. 7I). Western blotting and immunofluorescence analyses revealed that STAT3 knockdown led to significant accumulation of NRF2 in the cytoplasm, accompanied by a pronounced reduction in nuclear NRF2 levels (Fig. 7J), indicating impaired NRF2 nuclear translocation. Next, we investigated the effect of STAT3 knockdown on ferroptosis. Western blotting analysis of ferroptosis-related proteins showed that STAT3 silencing significantly increased the expression of ACSL4 and markedly reduced GPX4 expression in lenvatinib-resistant HCC cells (Fig. 7K), suggesting the induction of ferroptosis. Consistently, further assessment of redox- and ferroptosis-associated biomarkers demonstrated that STAT3 knockdown significantly elevated lipid peroxidation and lipid reactive oxygen species (ROS) levels (Figure S6D, Supporting Information), increased MDA levels (Fig. 7L), elevated intracellular ferrous iron (Fe²⁺) levels (Figure S6E, Supporting Information), and significantly decreased the GSH/GSSG ratio (Fig. 7M). Collectively, these results indicated that STAT3 silencing induces redox imbalance and ferroptosis in lenvatinib-resistant HCC cells. Taken together, our findings demonstrate that SERPINE2 activates the JAK2/STAT3 signaling pathway and that STAT3 interacts with NRF2 to facilitate NRF2 nuclear translocation.

### SERPINE2 activates the JAK2/STAT3 pathway to promote NRF2 nuclear translocation, induce GCLC transcription, suppress ferroptosis, and confer lenvatinib resistance in hepatocellular carcinoma

**Fig. 8:**
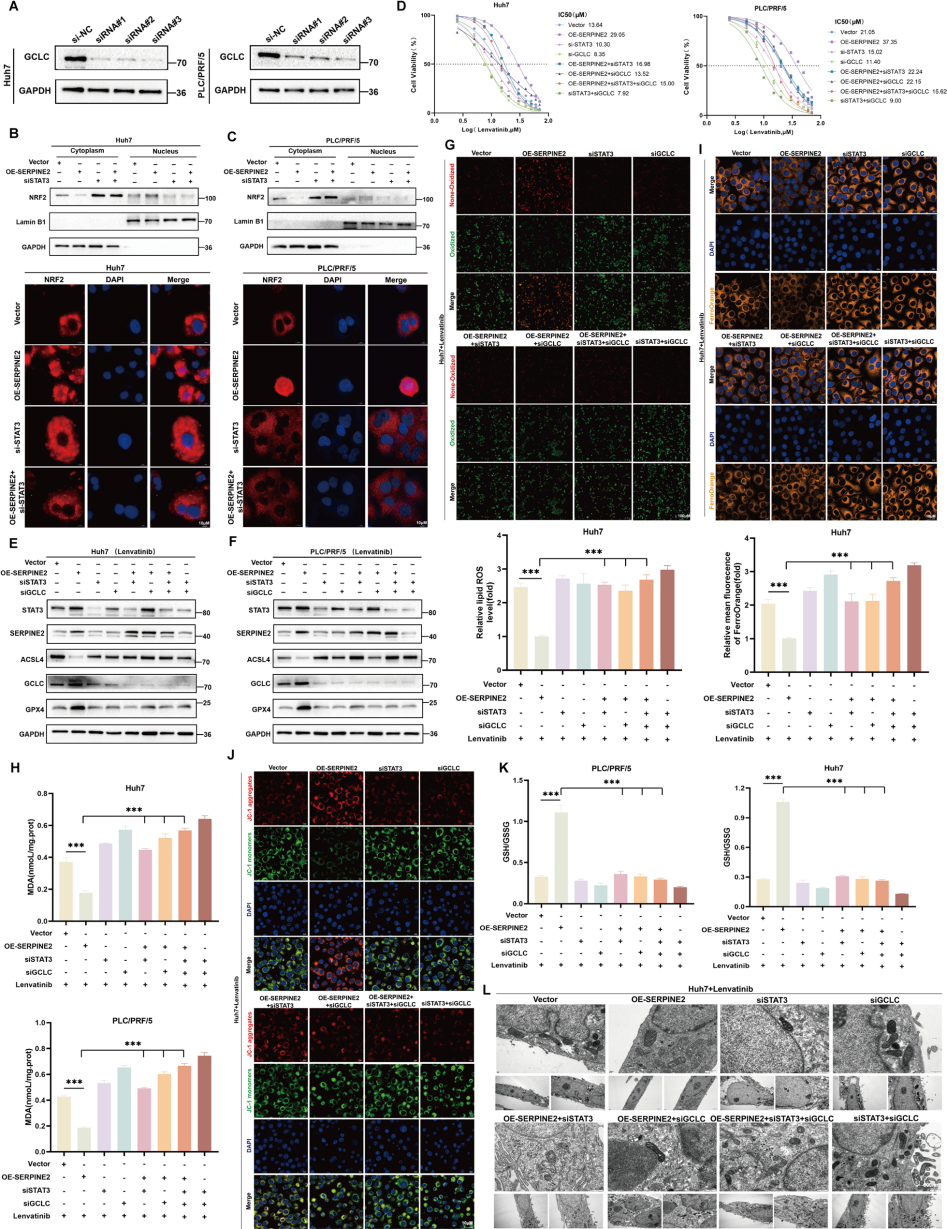
Rescue experiments validate that SERPINE2 suppresses ferroptosis and promotes lenvatinib resistance in hepatocellular carcinoma via the JAK2/STAT3–NRF2–GCLC axis. **A** In parental HCC cells overexpressing SERPINE2, GCLC-siRNAs were transfected and GCLC knockdown efficiency was verified by Western blotting; siRNA#3 with the highest knockdown efficiency was selected. **B**, **C** In parental cells overexpressing SERPINE2, STAT3 knockdown was performed, and NRF2 subcellular localization was analyzed by Western blotting and immunofluorescence. Nuclear NRF2 levels were not significantly increased, indicating that STAT3 depletion blocks SERPINE2-induced NRF2 nuclear translocation. **D** CCK-8 assays showed that SERPINE2 overexpression significantly increased the IC₅₀ of lenvatinib in parental cells, whereas additional knockdown of STAT3 or GCLC markedly restored lenvatinib sensitivity. **E**, **F** Under lenvatinib treatment, Western blot analysis of ferroptosis-related markers demonstrated that SERPINE2 overexpression rescued lenvatinib-induced ferroptotic phenotypes, while knockdown of STAT3 or GCLC effectively reversed SERPINE2-mediated ferroptosis resistance. **G**–**K** Analyses of redox and ferroptosis-associated parameters revealed that SERPINE2 overexpression suppressed increases in lipid ROS (**G**) and malondialdehyde (MDA) levels (**H**), reduced intracellular ferrous iron (Fe²⁺) accumulation (**I**), maintained mitochondrial membrane potential (ΔΨm) (**J**), and prevented a decrease in the GSH/GSSG ratio (**K**). These effects were significantly reversed by STAT3 or GCLC knockdown. **L** Transmission electron microscopy revealed mitochondrial ultrastructural changes. Lenvatinib treatment induced typical ferroptosis-associated mitochondrial damage (mitochondrial shrinkage, cristae reduction or loss, and membrane rupture), which was markedly alleviated by SERPINE2 overexpression; subsequent knockdown of STAT3 or GCLC re-induced mitochondrial injury. Data are presented as mean ± SD. Statistical analyses were performed using Student’s t test or one-way ANOVA. Statistical significance is indicated as follows: ns, not significant; *, *p* < 0.05; **, *p* < 0.01; ***, *p* < 0.001

To further investigate the roles of STAT3 and GCLC in the SERPINE2-mediated suppression of ferroptosis and induction of lenvatinib resistance in HCC, STAT3-specific small interfering RNA (STAT3-siRNA) and GCLC-specific siRNA (GCLC-siRNA) were transfected into parental HCC cells overexpressing SERPINE2. The knockdown efficiency of GCLC was validated by western blotting. siRNA#3, which exhibited the highest silencing efficiency, was selected for subsequent experiments (Fig. 8A).

We first examined whether STAT3 is required for the SERPINE2-mediated promotion of NRF2 nuclear translocation. STAT3 was silenced in parental HCC cells overexpressing SERPINE2. Western blotting and immunofluorescence analyses showed that nuclear NRF2 levels did not significantly increase following STAT3 knockdown (Fig. 8B–C), indicating that STAT3 silencing effectively blocked the regulatory effect of SERPINE2 on NRF2 nuclear translocation. Next, we investigated whether SERPINE2 regulates ferroptosis and induces lenvatinib resistance through STAT3 and GCLC. Overexpression of SERPINE2 in parental HCC cells significantly increased the IC_50_ of lenvatinib, whereas the knockdown of either STAT3 or GCLC markedly restored lenvatinib sensitivity in SERPINE2-overexpressing cells (Fig. 8D). Subsequent experiments were performed using lenvatinib treatment. Western blot analysis of ferroptosis-related marker proteins revealed that SERPINE2 overexpression rescued lenvatinib-induced ferroptosis, whereas knockdown of STAT3 or GCLC effectively reversed the ferroptosis resistance conferred by SERPINE2 overexpression (Fig. 8E–F). We further examined the redox- and ferroptosis-associated biomarkers. SERPINE2 overexpression markedly attenuated the increase in lipid ROS and MDA levels, prevented the elevation of intracellular ferrous iron (Fe²⁺) levels and mitochondrial membrane potential, and suppressed the decrease in GSH/GSSG ratio. In contrast, knockdown of STAT3 or GCLC reversed the protective effects of SERPINE2 overexpression, resulting in significantly increased lipid ROS (Fig. 8G and Figure S7A, Supporting Information) and MDA levels (Fig. 8H), elevated intracellular Fe²⁺ levels (Fig. 8I and Figure S7B, Supporting Information), mitochondrial membrane potential (Fig. 8J and Figure S7C, Supporting Information), and a marked reduction in the GSH/GSSG ratio (Fig. 8K). Transmission electron microscopy revealed that lenvatinib treatment induced characteristic mitochondrial alterations, including mitochondrial shrinkage, cristae loss or disappearance, and membrane rupture. These mitochondrial abnormalities were markedly alleviated by SERPINE2 overexpression, whereas STAT3 or GCLC knockdown abrogated the protective effects conferred by SERPINE2 overexpression (Fig. 8L and Figure S7D, Supporting Information). Collectively, these results demonstrate that SERPINE2 promotes STAT3–NRF2 interaction and facilitates NRF2 nuclear translocation, thereby regulating GCLC expression to suppress ferroptosis and ultimately induce lenvatinib resistance in hepatocellular carcinoma.

### Ruxolitinib and the NRF2 inhibitor ML385 restore lenvatinib sensitivity, overcome resistance, and enhance therapeutic efficacy in hepatocellular carcinoma

**Fig. 9:**
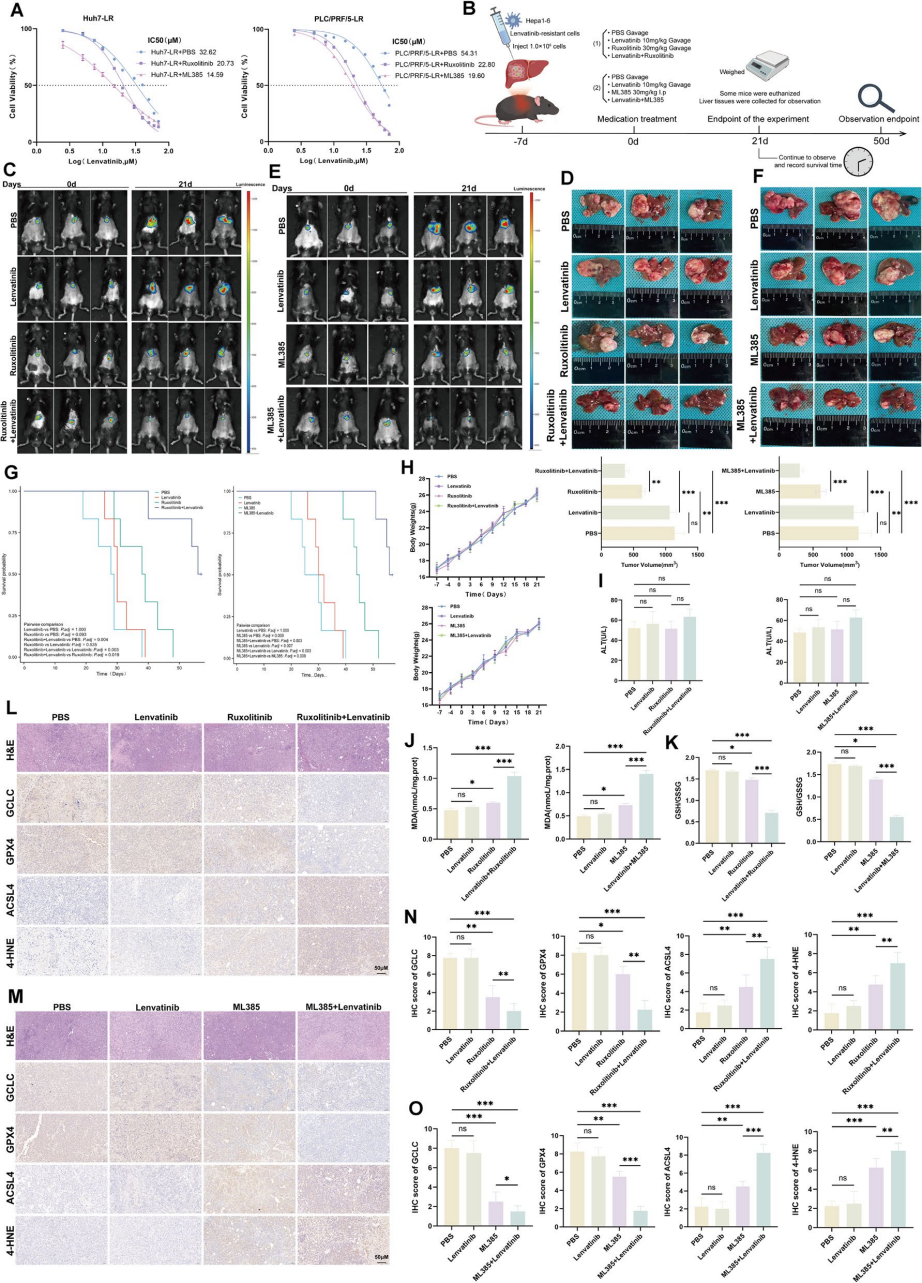
Evaluation of combination therapy targeting the SERPINE2–JAK/STAT–NRF2 pathway with lenvatinib in a mouse orthotopic hepatocellular carcinoma model to overcome drug resistance via ferroptosis. **A** CCK-8 assays showing that treatment with the JAK inhibitor ruxolitinib or the selective NRF2 inhibitor ML385 significantly reduced the IC₅₀ of lenvatinib in lenvatinib-resistant HCC cells. **B** Schematic illustration of the orthotopic liver cancer model established using lenvatinib-resistant Hepa1-6 cells and the corresponding treatment regimens. **C**, **D** Combination treatment with ruxolitinib and lenvatinib markedly suppressed tumor growth in the orthotopic HCC model, as evidenced by reduced tumor volume and bioluminescent signals. **E**, **F** ML385 combined with lenvatinib similarly enhanced the antitumor efficacy of lenvatinib and significantly inhibited tumor progression compared with monotherapy. **G** Kaplan–Meier survival analysis demonstrating that combination therapy with ruxolitinib or ML385 plus lenvatinib significantly prolonged overall survival compared with single-agent treatment. **H**, **I** Body weight monitoring and serum alanine aminotransferase (ALT) levels in each treatment group, showing no significant body weight loss or liver dysfunction, indicating good tolerability of the combination therapies in vivo. **J**, **K** Measurement of malondialdehyde (MDA) levels and GSH/GSSG ratios in tumor tissues, showing markedly increased lipid peroxidation and decreased antioxidant capacity in the combination treatment groups. **L**, **M** Representative immunohistochemical staining images of GPX4, ACSL4, GCLC, and the lipid peroxidation end product 4-hydroxynonenal (4-HNE) in tumor tissues. **N**, **O** Quantitative analysis of immunohistochemical staining showing that, compared with other treatment groups, combination therapy resulted in the lowest expression of GPX4 and GCLC and the highest levels of ACSL4 and 4-HNE, indicating robust activation of ferroptosis. Data are presented as mean ± SD. Statistical analyses were performed using Student’s t test or one-way analysis of variance (ANOVA). Statistical significance is indicated as follows: ns, not significant; *, *p* < 0.05; **, *p* < 0.01; ***, *p* < 0.001

After establishing the biological functions of SERPINE2, we evaluated the therapeutic potential of the JAK pathway inhibitor ruxolitinib and the selective NRF2 inhibitor ML385 to suppress ferroptosis and enhance lenvatinib sensitivity in HCC cells [28, 29]. As shown in Fig. 9A, treatment with Ruxolitinib or ML385 significantly reduced the IC_50_ of lenvatinib in lenvatinib-resistant HCC cells compared with control cells. To further assess the in vivo therapeutic efficacy of Ruxolitinib and ML385, a mouse orthotopic liver tumor model was established using lenvatinib-resistant Hepa1-6 cells. Drug administration was initiated seven days after tumor implantation, and the treatment regimen and grouping are illustrated in Fig. 9B. Compared to monotherapy, combination treatment with ruxolitinib (Fig. 9C–D) or ML385 (Fig. 9E–F) in conjunction with lenvatinib restored lenvatinib sensitivity, significantly enhanced antitumor efficacy, and resulted in markedly greater tumor regression. Notably, mice receiving combination therapy exhibited significantly prolonged overall survival compared to those receiving single-agent treatment or control therapy (Fig. 9G). In addition, no significant differences were observed in body weight changes or serum liver function parameters among the treatment groups (Fig. 9H–I), indicating the favorable tolerability of the combination regimens in vivo. Collectively, these findings demonstrate that, compared with monotherapy, combination treatment with Ruxolitinib or ML385 and lenvatinib effectively restored lenvatinib sensitivity in hepatocellular carcinoma, enhanced therapeutic efficacy, and exhibited good tolerability in vivo. 

To determine whether Ruxolitinib and ML385 enhance the therapeutic efficacy of lenvatinib in vivo by modulating ferroptosis and thereby overcoming lenvatinib resistance, we examined ferroptosis-related biomarkers and gene expression profiles in tumor tissues from each treatment group in mouse models. Compared to the monotherapy group, the combination treatment groups exhibited significantly higher levels of MDA (Fig. 9J) and a markedly reduced GSH/GSSG ratio (Fig. 9K), indicating enhanced lipid peroxidation and redox imbalance. IHC analyses were performed to evaluate the expression of ferroptosis-related genes, including GPX4, ACSL4, and GCLC, as well as the levels of the lipid peroxidation end-product 4-hydroxynonenal (4-HNE), in tumor tissues from each group (Fig. 9L–M). The results showed that compared with other treatment groups, tumors from the combination therapy groups exhibited the lowest expression levels of GPX4 and GCLC, whereas the expression levels of ACSL4 and 4-HNE were the highest (Fig. 9N–O). Furthermore, transmission electron microscopy analysis of in vivo tumor tissues revealed that the combination treatment induced classic ferroptotic mitochondrial alterations, including mitochondrial shrinkage, increased mitochondrial membrane density, and loss of mitochondrial cristae. In contrast, tumors from the control or monotherapy groups exhibited relatively normal or only mildly altered mitochondrial morphology (Figure S8A-B, Supporting Information). Collectively, these findings demonstrate that in preclinical hepatocellular carcinoma models, combination treatment with Ruxolitinib or an NRF2 inhibitor and lenvatinib restores lenvatinib sensitivity by promoting ferroptosis, thereby significantly enhancing the antitumor efficacy of lenvatinib in vivo.

### SERPINE2 expression positively correlates with GCLC expression in hepatocellular carcinoma, and their co-overexpression predicts poor prognosis

**Fig. 10:**
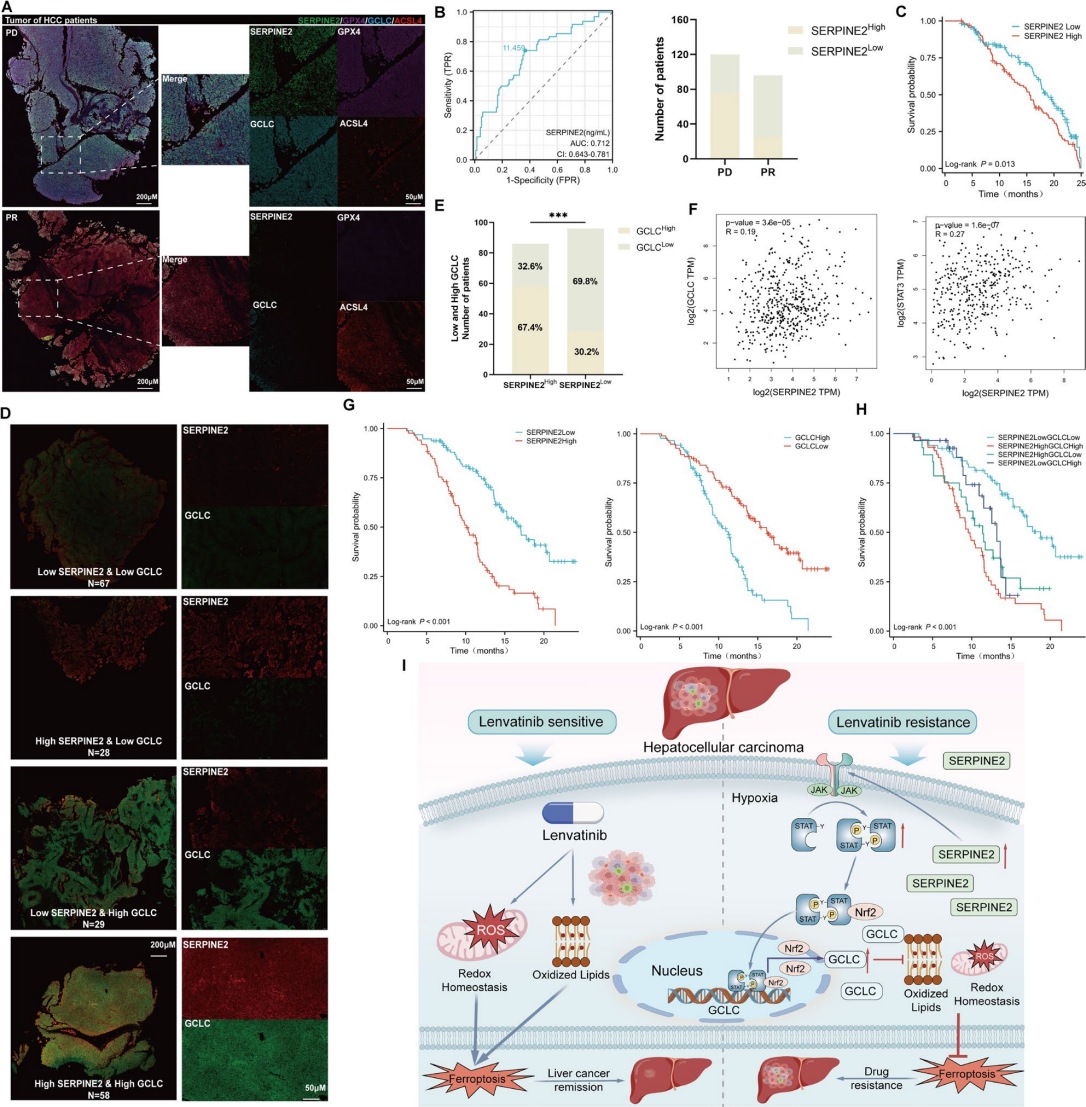
Clinical validation of the association between SERPINE2 and GCLC with ferroptosis and their prognostic value in lenvatinib resistance in hepatocellular carcinoma. **A** Multiplex immunofluorescence analysis showed that, compared with PR (partial response) patients, PD (progressive disease) patients exhibited elevated SERPINE2 expression accompanied by increased GCLC and GPX4 levels and decreased ACSL4 expression, consistent with the molecular mechanism of SERPINE2 identified in this study. **B** Serum SERPINE2 protein concentrations were measured by ELISA in peripheral blood samples from 216 patients with hepatocellular carcinoma, and patients were stratified into SERPINE2 high- and low-expression groups according to the ROC curve AUC cutoff value of 11.459 ng/mL. **C** Kaplan–Meier analysis showed that the overall survival (OS) of patients in the SERPINE2 high-expression group was significantly shorter than that of the low-expression group (*p* < 0.05), indicating the potential of SERPINE2 as a circulating biomarker for lenvatinib resistance in HCC. **D** In tumor tissue paraffin sections from 182 HCC patients treated with lenvatinib, immunofluorescence staining was performed to detect SERPINE2 and GCLC expression. Based on expression levels, patients were classified into four groups: Group I (low SERPINE2/low GCLC), Group II (high SERPINE2/low GCLC), Group III (low SERPINE2/high GCLC), and Group IV (high SERPINE2/high GCLC). **E** Immunofluorescence analysis demonstrated that the proportion of GCLC-high tumors was significantly higher in SERPINE2-high tumors than in SERPINE2-low tumors. **F** Analysis of the TCGA hepatocellular carcinoma cohort revealed significant positive correlations between SERPINE2 transcript levels and STAT3 as well as GCLC expression. **G** Kaplan–Meier survival analysis showed that patients with high SERPINE2 and GCLC expression in tumor tissues had significantly shorter overall survival than those with low expression. **H** Kaplan–Meier survival analysis of the four patient groups indicated that Group IV (high SERPINE2/high GCLC) had the poorest prognosis, whereas Group I (low SERPINE2/low GCLC) had the most favorable survival outcome. **I** Schematic model summarizing the proposed mechanism: under the hypoxic liver tumor microenvironment, SERPINE2 is upregulated and activates the JAK2/STAT3 signaling pathway, leading to increased STAT3 levels. STAT3 interacts with NRF2 and promotes its nuclear translocation, thereby enhancing NRF2-dependent transcription of GCLC, suppressing lipid peroxidation, blocking ferroptosis, and ultimately conferring lenvatinib resistance in hepatocellular carcinoma cells. Data are presented as mean ± SD. Statistical analyses were performed using Student’s t-test or one-way ANOVA. Statistical significance is indicated as follows: ns, not significant; *, *p* < 0.05; **, *p* < 0.01; ***, *p* < 0.001

We further validated the role of SERPINE2 in regulating ferroptosis and its association with lenvatinib resistance in HCC specimens. Multiplex immunofluorescence analysis revealed that compared with patients achieving PR, tumors from patients with PD exhibited markedly elevated SERPINE2 expression, accompanied by increased expression of GCLC and GPX4 and decreased expression of ACSL4 (Fig. 10A). These expression patterns were consistent with the molecular mechanism of SERPINE2-mediated ferroptosis suppression identified in this study. Given that SERPINE2 is a secreted protein, we evaluated its potential as a circulating biomarker for predicting lenvatinib resistance. Peripheral blood samples were collected from 216 HCC patients receiving lenvatinib treatment, and serum SERPINE2 protein levels were quantified using enzyme-linked immunosorbent assay (ELISA). Based on treatment response and receiver operating characteristic (ROC) curve analysis, patients were stratified into SERPINE2 high- and low-expression groups using an optimal cut-off value of 11.459 ng/mL. Among the 102 patients in the SERPINE2 high-expression group, 76 experienced progressive disease, whereas 70 of the 114 patients in the SERPINE2 low-expression group achieved partial response (Fig. 10B). Survival analysis further demonstrated that patients with high serum SERPINE2 levels had significantly shorter overall survival compared with those in the low-expression group (Fig. 10C), consistent with the tissue-based findings shown in Fig. 3I–J. Collectively, these results indicate that SERPINE2 has strong potential as a circulating biomarker for predicting lenvatinib resistance and clinical outcomes in patients with HCC.

To further investigate the correlation between SERPINE2 and GCLC expression in HCC, tumor tissue paraffin blocks were collected from 182 HCC patients who received lenvatinib treatment. Multiplex immunofluorescence staining was performed to evaluate the protein expression of SERPINE2 and GCLC. Based on the expression patterns, patients were stratified into four groups (Fig. 10D):Group I (*n* = 67), low SERPINE2 and low GCLC expression; Group II (*n* = 28), high SERPINE2 and low GCLC expression; Group III (*n* = 29), low SERPINE2 and high GCLC expression; and Group IV (*n* = 58), high SERPINE2 and high GCLC expression. As shown in Fig. 10E, the proportion of tumors with high GCLC expression was significantly higher in SERPINE2-high tumors than that in SERPINE2-low tumors. Consistently, analysis of TCGA liver hepatocellular carcinoma (LIHC) cohort demonstrated that SERPINE2 transcript levels were significantly and positively correlated with those of STAT3 and GCLC (Fig. 10F). Kaplan–Meier survival analysis further revealed that patients with high SERPINE2 expression in tumor tissues exhibited significantly shorter OS than those with low SERPINE2 expression (Fig. 10G), consistent with our previous findings. Similarly, patients with high GCLC expression had significantly poorer OS than those with low GCLC expression. Notably, among the four patient subgroups, Group IV (high SERPINE2 and GCLC expression) exhibited the shortest OS, whereas Group I (low SERPINE2 and GCLC expression) demonstrated the most favorable survival outcomes (Fig. 10H). Collectively, these results indicate that SERPINE2 and GCLC expression levels are positively correlated in hepatocellular carcinoma, and that their co-overexpression may serve as a robust prognostic biomarker for predicting lenvatinib resistance and poor clinical outcomes in HCC patients.

## Discussion

Hepatocellular carcinoma (HCC) remains one of the leading causes of cancer-related mortality worldwide, with limited therapeutic options available for patients with advanced disease. Although multitarget tyrosine kinase inhibitors (TKIs) such as lenvatinib have improved clinical outcomes in a subset of patients, the rapid development of acquired resistance severely restricts their long-term efficacy [30, 31]. Elucidating the molecular basis of lenvatinib resistance and identifying tractable targets represent critical challenges in HCC precision therapy. In this study, we identified SERPINE2 as a key mediator of lenvatinib resistance by integrating hypoxia-driven and lenvatinib-resistant HCC models. SERPINE2 is markedly upregulated in resistant tumors and is closely associated with a poor therapeutic response and unfavorable patient survival. Importantly, we revealed a previously unrecognized function of SERPINE2 in HCC: it promotes tumor progression and confers lenvatinib resistance by suppressing ferroptosis. Mechanistically, SERPINE2 activated the JAK2/STAT3 signaling cascade, leading to enhanced STAT3 activation and its interaction with NRF2. This interaction facilitates NRF2 nuclear translocation and the transcriptional activation of GCLC, a rate-limiting enzyme in glutathione biosynthesis. The upregulation of GCLC reinforces antioxidant capacity, inhibits lipid peroxidation and ferroptotic cell death, and enables HCC cells to survive under lenvatinib-induced oxidative stress, ultimately driving drug resistance (Fig. 10I). Notably, this study is the first to systematically delineate the SERPINE2–JAK2/STAT3–NRF2–GCLC axis as a central regulator of ferroptosis resistance in HCC. Clinically, SERPINE2 expression in tumor tissues and peripheral blood correlates with treatment response and prognosis, supporting its potential utility as a predictive biomarker for lenvatinib efficacy. Moreover, the pharmacological inhibition of JAK/STAT signaling or NRF2 activity effectively restores ferroptosis and sensitizes HCC cells to lenvatinib in preclinical models, underscoring the translational relevance of targeting this pathway. Collectively, our findings expand the functional landscape of SERPINE2 in HCC and provide mechanistic insights into ferroptosis-mediated lenvatinib resistance. Targeting the SERPINE2-driven antioxidant program may represent a promising strategy for overcoming therapeutic resistance and improving clinical outcomes in HCC.

SERPINE2, also known as protease nexin-1 (PN-1), is a secreted serine protease inhibitor that has been primarily studied for its role in extracellular matrix remodeling, tumor invasion, and metastasis [32–34]. For example, Liu et al. demonstrated that SERPINE2 mediates a positive feedback loop between tumor cells and M2 macrophages, thereby accelerating colorectal cancer progression [35]. Zhang et al. reported that SERPINE2 promotes the metastasis of esophageal squamous cell carcinoma through the activation of BMP4 signaling [36]. In addition, SERPINE2 has been implicated in radioresistance in lung cancer; silencing SERPINE2 suppresses the accumulation of p-ATM and the downstream DNA repair protein RAD51 during DNA damage repair, whereas restoration of RAD51 rescues DNA repair capacity and radioresistant phenotypes [37]. Consistently, SERPINE2 knockdown enhanced radiosensitivity both in vitro and in vivo. Previous studies have shown that SERPINE2 promotes HCC metastasis by inhibiting EGFR degradation [19]. However, its role in resistance to targeted therapies has not been systematically elucidated. In the present study, we established, for the first time, a direct link between SERPINE2 and lenvatinib resistance in HCC, identifying SERPINE2 as a critical molecular node connecting the hypoxic tumor microenvironment with acquired drug resistance. Through integrative transcriptomic analyses of hypoxia-adapted and lenvatinib-resistant HCC models, SERPINE2 has emerged as a commonly upregulated key driver gene under both conditions. Functional assays further demonstrated that SERPINE2 markedly enhances HCC cell proliferation, suppresses apoptosis, and significantly increases the IC₅₀ of lenvatinib, whereas genetic or pharmacological inhibition of SERPINE2 robustly restores drug sensitivity in both in vitro and in vivo models. Collectively, these findings indicate that SERPINE2 is a potent oncogenic factor that promotes malignant progression and drives lenvatinib resistance in hepatocellular carcinoma.

Ferroptosis is a regulated form of cell death characterized by iron-dependent lipid peroxidation, and has recently been recognized as a critical mechanism underlying the therapeutic efficacy of various anticancer strategies, including targeted therapy and immunotherapy. Accumulating evidence indicates that acquisition of ferroptosis resistance by tumor cells is a major cause of treatment failure [15, 38]. Recently, Tian et al. reported that hnRNPA2B1-regulated tumor-derived exosomal miR-214-3p promotes cisplatin resistance in osteosarcoma by suppressing ferroptosis [39]. Similarly, other studies have demonstrated that the long noncoding RNA LINC00152 confers tamoxifen resistance in breast cancer by blocking tamoxifen-induced ferroptosis [40]. Guo et al. further revealed that PIP5K1A competitively binds to the Kelch domain of Kelch-like ECH-associated protein 1 (KEAP1) in a kinase-independent manner, allowing NRF2 to escape ubiquitination-mediated degradation, thereby promoting NRF2-dependent transcriptional programs that suppress ferroptosis and drive sorafenib resistance in hepatocellular carcinoma [41]. In the present study, integrative multi-omics analyses combining transcriptomic and proteomic profiling revealed that knockdown of SERPINE2 in lenvatinib-resistant HCC cells resulted in robust activation of ferroptosis-related pathways, particularly those involved in glutathione metabolism, cysteine metabolism, and cellular redox homeostasis. Among these pathways, we further identified the glutamate–cysteine ligase catalytic subunit (GCLC) as the most critical downstream regulator of ferroptosis controlled by SERPINE2, highlighting its central biological significance in mediating ferroptosis resistance and therapeutic insensitivity.

GCLC is the rate-limiting enzyme in glutathione (GSH) biosynthesis, and its activity directly determines intracellular GSH availability. The GSH–GPX4 axis constitutes a critical defense system for detoxifying lipid peroxides and preventing ferroptotic cell death [42, 43]. Previous studies have shown that upregulation of GCLC markedly enhances tumor cell survival under oxidative stress and is closely associated with resistance to multiple anticancer therapies [17, 44]. However, in HCC, particularly under hypoxic conditions and lenvatinib-induced therapeutic pressure, the upstream regulatory mechanisms governing GCLC expression have remained largely unexplored. Through integrative multi-omics analyses and functional assays, our study demonstrated that SERPINE2 promotes ferroptosis resistance by enhancing the transcriptional expression of GCLC, thereby significantly increasing intracellular GSH levels and sustaining GPX4 activity. This mechanism effectively suppressed lipid peroxidation and ferroptotic cell death, providing a molecular explanation for how SERPINE2-high HCC cells maintain redox homeostasis during lenvatinib treatment. Functionally, these findings establish GCLC as a pivotal node linking tumor metabolic reprogramming, antioxidant defense, and resistance to targeted therapy. Notably, beyond its canonical role in glutathione synthesis, emerging evidence suggests that GCLC modulates cysteine metabolic flux, indirectly influencing iron homeostasis and mitochondrial function, thereby exerting broader regulatory effects on ferroptosis [45]. Consistent with this notion, we observed preserved mitochondrial membrane potential and ultrastructural integrity in SERPINE2-overexpressing cells, further supporting the central role of GCLC in SERPINE2-mediated ferroptosis suppression. Clinically, correlation analyses revealed a significant positive association between GCLC and SERPINE2 expression in HCC tissues, and patients with concurrent high expression of both genes exhibited the poorest overall survival, underscoring the potential prognostic value of GCLC beyond its role as a downstream effector. In line with these observations, we further confirmed that SERPINE2 overexpression significantly reduced intracellular lipid reactive oxygen species, MDA, and Fe²⁺ levels while preserving mitochondrial membrane potential and ultrastructural integrity, thereby effectively inhibiting ferroptosis. Conversely, genetic or pharmacological inhibition of SERPINE2 or GCLC robustly induced ferroptotic cell death and restored lenvatinib sensitivity, whereas the ferroptosis inhibitor, ferrostatin-1, partially reversed these effects. Collectively, these findings provide compelling evidence that ferroptosis is a core mechanism underlying SERPINE2-mediated lenvatinib resistance in HCC.

Given that SERPINE2 is predominantly localized in the cytoplasm and does not function as a transcription factor, we investigated the upstream mechanisms by which SERPINE2 regulates GCLC transcription. Nuclear factor erythroid 2–related factor 2 (NRF2) is a master transcription factor responsible for maintaining cellular redox homeostasis and protecting cells against oxidative stress, and GCLC is one of its canonical downstream target genes whose activity is subject to multilayered and tightly regulated control [46]. Under basal conditions, NRF2 activity is strictly restrained by KEAP1–CUL3–mediated ubiquitin–proteasome degradation. Oxidative or electrophilic stress disrupts the KEAP1–NRF2 interaction, leading to NRF2 stabilization and nuclear translocation, where it activates the transcription of antioxidant and ferroptosis-suppressive genes, including GCLC, SLC7A11, and GPX4 [47]. Recent studies have demonstrated that in addition to the classical KEAP1-dependent regulatory mechanism, NRF2 can also be activated through KEAP1-independent pathways, such as regulation via the GSK-3β/β-TrCP axis, oncogenic signaling–mediated protein stabilization, and cooperative interactions with other transcription factors that facilitate its nuclear translocation [48, 49]. In hepatocellular carcinoma and other solid tumors, sustained NRF2 activation has been widely implicated in ferroptosis resistance and resistance to targeted therapies. NRF2 suppresses ferroptosis through multiple mechanisms, including the upregulation of GCLC and SLC7A11 to enhance glutathione biosynthesis, maintenance of GPX4 activity to detoxify lipid peroxides, and modulation of iron metabolism–related genes to reduce labile iron pools [50–53]. Accordingly, NRF2 is recognized as a central hub that links oxidative stress adaptation, metabolic reprogramming, and therapeutic resistance. In this study, we demonstrated that SERPINE2 does not directly affect NRF2 mRNA expression or total protein levels but instead markedly promotes NRF2 nuclear translocation. Mechanistically, SERPINE2 activates the JAK2/STAT3 signaling pathway, enhances STAT3 phosphorylation, and facilitates the interaction between STAT3 and NRF2, thereby cooperatively promoting NRF2 nuclear accumulation and transcriptional activation. Silencing STAT3 effectively abrogated SERPINE2-mediated NRF2 nuclear translocation, downregulated GCLC expression, and reactivated ferroptosis, indicating that STAT3 serves as a critical molecular bridge within this signaling axis. Notably, this regulatory mode is distinct from the classical KEAP1-dependent mechanism of NRF2 stabilization and reveals a novel KEAP1-independent NRF2 activation paradigm driven by oncogenic signaling–mediated protein–protein interactions. This finding further expands our understanding of the complexity of NRF2 regulatory networks in cancers.

From a therapeutic and translational perspective, our findings further highlight the critical role of the SERPINE2–STAT3–NRF2–GCLC axis in lenvatinib resistance in HCC. We demonstrated that the JAK inhibitor ruxolitinib and the selective NRF2 inhibitor ML385 effectively restored ferroptosis and that their combination with lenvatinib overcame acquired resistance and significantly enhanced the antitumor efficacy of lenvatinib. In vivo, the combination treatment markedly increased lipid peroxidation, upregulated ACSL4 and 4-hydroxynonenal (4-HNE) expression, and downregulated GCLC and GPX4 levels, resulting in pronounced tumor growth inhibition and prolonged survival without evident systemic toxicity. These results provide compelling experimental evidence supporting the strategy of targeting ferroptosis resistance pathways to overcome lenvatinib resistance. We further confirmed a significant positive association between SERPINE2 and GCLC expression in HCC tissues, and patients with concomitant high expression of both markers exhibited the poorest overall survival, underscoring the prognostic relevance of this signaling axis. Notably, this study is the first to validate the feasibility of SERPINE2 as a potential liquid biopsy biomarker. Serum SERPINE2 levels were significantly elevated in patients with lenvatinib-resistant disease and effectively discriminated between responders and those with disease progression. These findings suggest that SERPINE2 is not only a functional mediator of therapeutic resistance, but may also serve as a clinically informative biomarker to guide lenvatinib treatment decisions and the selection of combination strategies. Collectively, our study establishes a comprehensive evidence framework spanning mechanistic insight, therapeutic response, and clinical prediction, supporting SERPINE2–GCLC–directed combinatorial interventions as promising avenues for overcoming lenvatinib resistance and advancing personalized therapy in HCC.

Despite comprehensive elucidation of the novel mechanism by which SERPINE2 suppresses ferroptosis and promotes lenvatinib resistance, several limitations should be acknowledged. As a secreted protein, SERPINE2 may exert paracrine effects on other cell types within the tumor microenvironment, such as immune cells and cancer-associated fibroblasts. Whether SERPINE2 further modulates ferroptosis sensitivity through regulation of the tumor immune microenvironment warrants further investigation. For instance, Sun et al. demonstrated through single-cell transcriptomic analyses that SERPINE2 secreted by cancer-associated fibroblasts (CAFs) in gastric cancer is closely associated with an immunosuppressive tumor microenvironment and resistance to immunotherapy, identifying CAF-derived SERPINE2 as a promising immuno-oncology target with a potential therapeutic value in combination with immunotherapy [54]. Given that current treatment paradigms for hepatocellular carcinoma increasingly emphasize combinations of targeted therapy and immunotherapy, it remains to be determined whether SERPINE2 inhibition could synergize with first-line immune checkpoint blockade to further enhance therapeutic efficacy and improve clinical outcomes in HCC patients. In addition, the safety profile, optimal dosing strategies, and clinical feasibility of SERPINE2-targeted combination therapies require validation through well-designed prospective clinical studies. Addressing these issues is essential for translating the mechanistic insights uncovered in this study into effective and clinically applicable therapeutic strategies.

## Conclusion

In summary, this is the first study to elucidate the mechanistic role of SERPINE2 in the acquisition of lenvatinib resistance in hepatocellular carcinoma. We identified a previously unrecognized SERPINE2–JAK2/STAT3–NRF2–GCLC signaling axis and systematically delineated how SERPINE2 promotes lenvatinib resistance by suppressing ferroptosis in HCC. These findings not only expand the functional landscape of SERPINE2 in liver cancer biology, but also provide a novel theoretical framework and potential therapeutic targets for overcoming lenvatinib treatment failure by targeting ferroptosis resistance, ultimately offering new opportunities to improve clinical outcomes in patients with hepatocellular carcinoma.

## Supplementary Information

Supplementary Material 1.

Supplementary Material 2.

## Data Availability

The datasets used and/or analysed during the current study are available from the corresponding author on reasonable request.

## Abbreviations

4-HNE: 4-hydroxynonenal
ACSL4: Acyl-CoA synthetase long-chain family member 4
ALT: Alanine aminotransferase
ANOVA: Analysis of variance
AREs: Antioxidant response elements
AUC: Area under the curve
CAFs: Cancer-associated fibroblasts
CCK-8: Cell Counting Kit-8
Co-IP: Co-immunoprecipitation
CR: Complete response
DEGs: Differentially expressed genes
DIA: Data-independent acquisition
DMEM: Dulbecco’s Modified Eagle’s Medium
ECL: Enhanced chemiluminescence
EGFR: Epidermal growth factor receptor
ELISA: Enzyme-linked immunosorbent assay
FDRs: False discovery rates
Fe²⁺: Ferrous iron
Fer-1: Ferrostatin-1
GCLC: Glutamate–cysteine ligase catalytic subunit
GO: Gene Ontology
GPX4: Glutathione peroxidase 4
GSEA: Gene set enrichment analysis
GSH/GSSG: Reduced/oxidized glutathione
HCC: Hepatocellular carcinoma
HIF-1α: Hypoxia-inducible factor-1α
IC₅₀: Half-maximal inhibitory concentration
IF: Immunofluorescence
IHC: Immunohistochemical
KEAP1: Kelch-like ECH-associated protein 1
LIHC: Liver Hepatocellular Carcinoma
Lipid ROS: Lipid reactive oxygen species
logFC: log₂ fold change
MDA: Malondialdehyde
mRECIST: Modified Response Evaluation Criteria in Solid Tumors
Nano-LC: Nano–liquid chromatography
NRF2: Nuclear factor erythroid 2–related factor 2
ORR: Objective response rate
OS: Overall survival
PD: Progressive disease
PFS: Progression-free survival
PI: Propidium iodide
PN-1: Protease nexin-1
PR: Partial response
p-JAK2 / p-STAT3: Phosphorylated JAK2 / Phosphorylated STAT3
RNA-seq: RNA sequencing / transcriptome sequencing
ROC: Receiver operating characteristic
ROIs: Regions of interest
RT–qPCR: Reverse transcription quantitative PCR
SD: Standard deviation
SERPINE2: Serine protease inhibitor E family member 2
shRNAs: Short hairpin RNAs
siRNAs: Small interfering RNAs
SPF: Specific pathogen-free
STAT3: Signal transducer and activator of transcription 3
TCGA: The Cancer Genome Atlas
TEM: Transmission electron microscopy
TIMER: Tumor Immune Estimation Resource
TKIs: Tyrosine kinase inhibitors
ΔΨm: Mitochondrial membrane potential
