# Supplementary material for: SERPINE2-mediated activation of JAK2/STAT3 facilitates NRF2 nuclear translocation and GCLC transcription to confer ferroptosis resistance and lenvatinib resistance in hepatocellular carcinoma
Source: J Exp Clin Cancer Res. 2026 Jun 6;45:174. doi: 10.1186/s13046-026-03746-y (PMC13459319; doi:10.1186/s13046-026-03746-y)
Supplement: Supplementary file 1 [file 13046_2026_3746_MOESM1_ESM.pdf]

SERPINE2-mediated activation of JAK2/STAT3 facilitates NRF2 nuclear translocation and GCLC transcription to confer ferroptosis resistance and lenvatinib resistance in hepatocellular carcinoma

Kan Liu[#], Shenglan Huang[#], Yongkang Xu, Ye Mao, Ning Chen, Yixin Yuan, Qinwen Xiao, Shumin Fu*, Jianbing Wu*

Kan Liu[#]

Department of Oncology, The Second Affiliated Hospital, Jiangxi Medical College, Nanchang University, Nanchang, Jiangxi Province, P.R. China.

E-mail:15179610710@163.com

Shenglan Huang[#]

Department of Oncology, The Second Affiliated Hospital, Jiangxi Medical College, Nanchang University, Nanchang, Jiangxi Province, P.R. China.

E-mail: 0796643011@163.com

Yongkang Xu

Department of Oncology, The Second Affiliated Hospital, Jiangxi Medical College, Nanchang University, Nanchang, Jiangxi Province, P.R. China.

E-mail: ykxu666@163.com

Ye Mao

Department of Oncology, The Second Affiliated Hospital, Jiangxi Medical College, Nanchang University, Nanchang, Jiangxi Province, P.R. China.

E-mail:rayooocat111@163.com

Ning Chen

Department of Oncology, The Second Affiliated Hospital, Jiangxi Medical College, Nanchang University, Nanchang, Jiangxi Province, P.R. China.

E-mail: chenning942668@163.com

Yixin Yuan

Department of Oncology, The Second Affiliated Hospital, Jiangxi Medical College, Nanchang University, Nanchang, Jiangxi Province, P.R. China.

E-mail: yuanyixin236@163.com

Qinwen Xiao

Department of Oncology, The Second Affiliated Hospital, Jiangxi Medical College, Nanchang

University, Nanchang, Jiangxi Province, P.R. China.

E-mail: 3589008370@qq.com

Shumin Fu*

Department of Oncology, The Second Affiliated Hospital, Jiangxi Medical College, Nanchang University, Nanchang, Jiangxi Province, P.R. China.

E-mail: min_111222@163.com

Jianbing Wu*

Department of Oncology, The Second Affiliated Hospital, Jiangxi Medical College, Nanchang University, Nanchang, Jiangxi Province, P.R. China.

E-mail: hhgwjb@163.com

Address: No. 1, Minde Road, Donghu District, Nanchang City, Jiangxi Province, China

# Contributed equally   * corresponding author

Keywords: Hepatocellular carcinoma, Lenvatinib resistance, Ferroptosis, SERPINE2, STAT3, GCLC

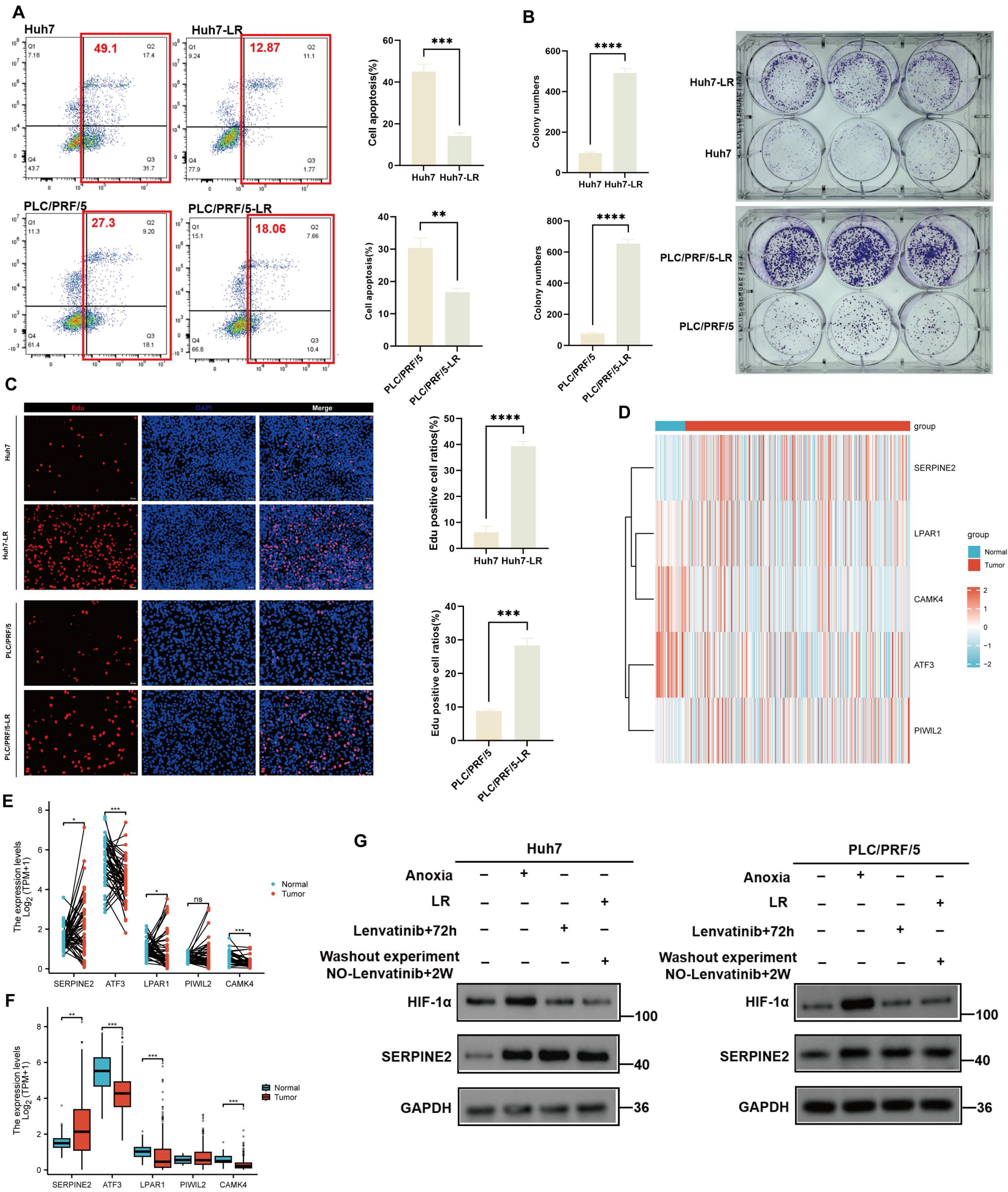

Figure S1. Functional validation of lenvatinib-resistant HCC cell models and identification of SERPINE2 as a key candidate gene. A) Flow cytometric analysis of apoptosis in parental and lenvatinib-resistant (LR) Huh7 and PLC/PRF/5 cells treated with the same concentration of lenvatinib, showing reduced apoptosis in LR cells. B) Colony formation assays comparing parental and LR Huh7 and PLC/PRF/5 cells under lenvatinib treatment, indicating that the clonogenic capacity of LR cells is largely unaffected by lenvatinib. C) EdU incorporation assays assessing cell proliferation in parental and LR Huh7 and PLC/PRF/5 cells treated with lenvatinib, demonstrating that proliferative activity of LR

cells is minimally affected by lenvatinib. D – F) Bioinformatic analyses of candidate genes (SERPINE2, ATF3, LPAR1, PIWIL2 and CAMK4) using the TCGA-LIHC cohort, including their expression patterns in tumor versus normal tissues and their associations with patient prognosis. G) Evaluation of SERPINE2 expression in parental HCC cells (Huh7 and PLC/PRF/5) treated with lenvatinib at the IC_{50} concentration for 48 h under normoxic conditions, and in lenvatinib-resistant cells (Huh7-LR and PLC/PRF/5-LR) cultured in a lenvatinib-free medium for 14 days (washout experiment). Protein level of SERPINE2 were determined by Western blotting. Data are presented as mean ± SD. Statistical analyses were performed using Student's t-test or one-way ANOVA, as appropriate. Statistical significance is indicated as ns, not significant; *, $p < 0.05$; **, $p < 0.01$; ***, $p < 0.001$.

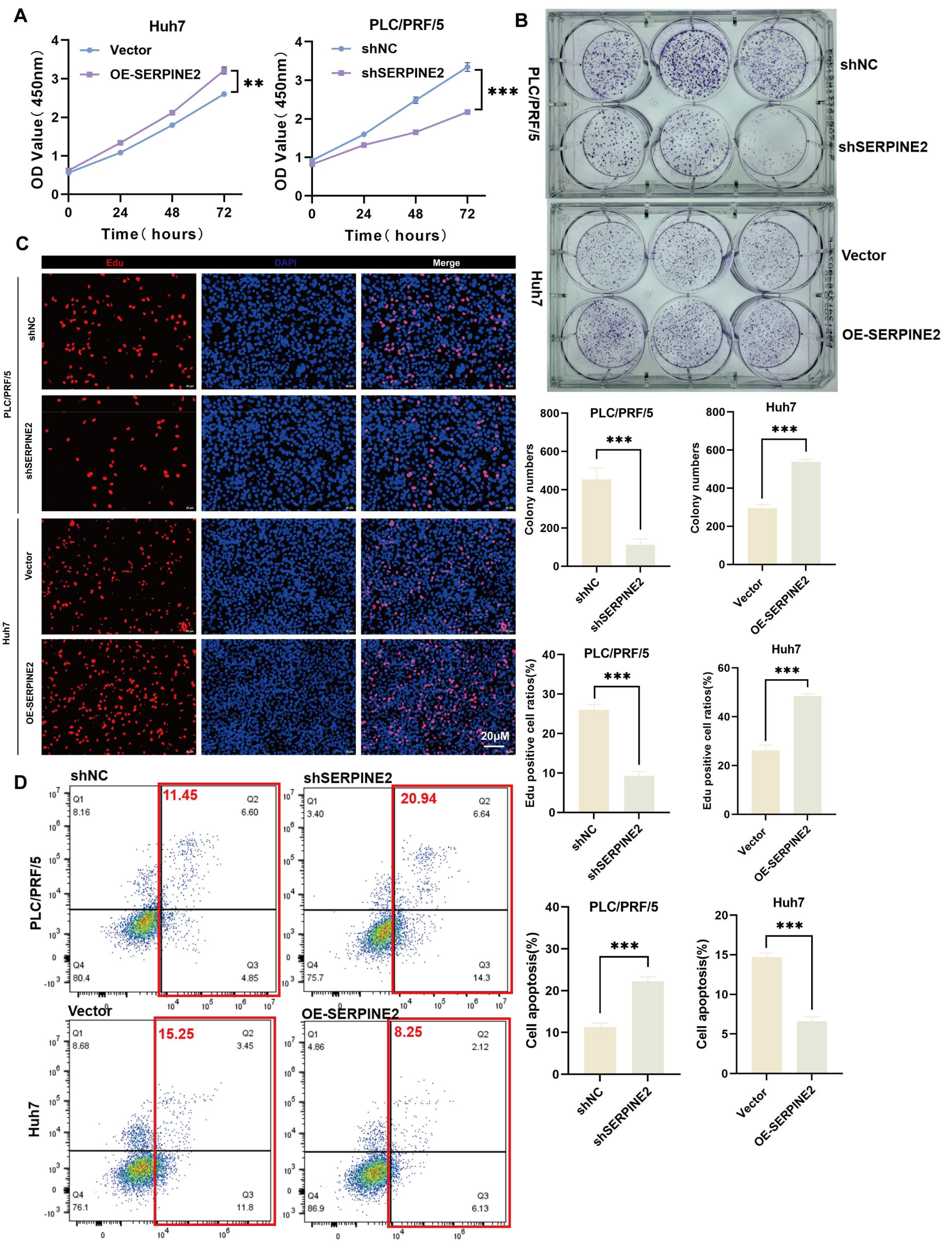

Figure S2. SERPINE2 promotes hepatocellular carcinoma cell proliferation and survival in vitro. A) CCK-8 assays showing cell viability curves of Huh7 and PLC/PRF/5 cells following SERPINE2 knockdown or overexpression at the indicated time points. B) Representative images and quantitative analysis of colony formation assays in Huh7 and PLC/PRF/5 cells with SERPINE2 knockdown or overexpression. C) Representative immunofluorescence images and quantification of EdU incorporation in Huh7 and

PLC/PRF/5 cells after SERPINE2 silencing or ectopic expression. D) Flow cytometric analysis and statistical quantification of apoptotic cell populations in Huh7 and PLC/PRF/5 cells with SERPINE2 knockdown or overexpression.Data are presented as mean ± SD from at least three independent experiments. Statistical analyses were performed using Student's t-test or one-way ANOVA. Statistical significance is indicated as follows: ns, not significant; *, $p < 0.05$; **, $p < 0.01$; ***, $p < 0.001$.

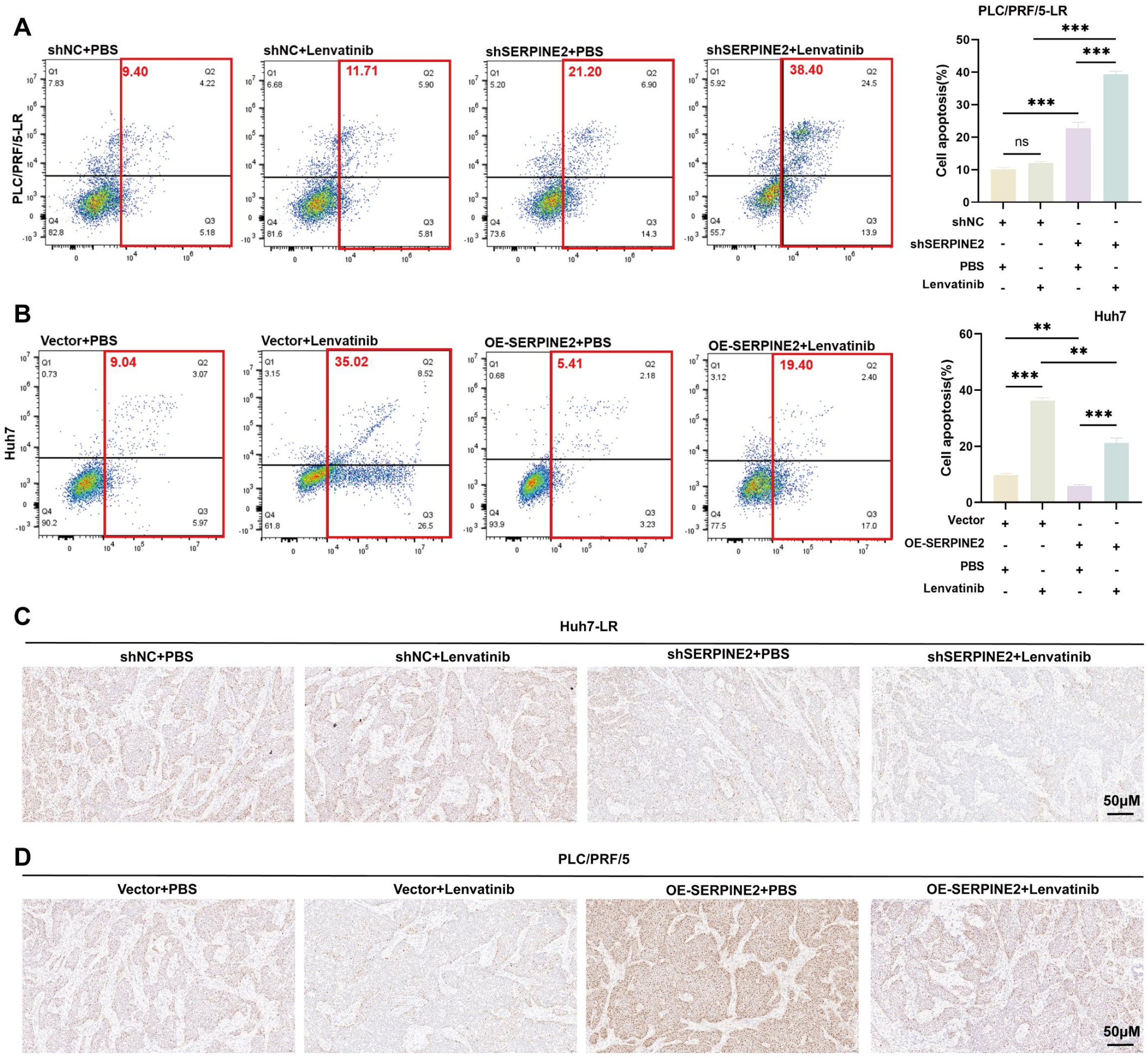

Figure S3. SERPINE2 regulates lenvatinib-induced apoptosis and tumor growth in hepatocellular carcinoma. A) Flow cytometric analysis of apoptosis in lenvatinib-treated PLC/PRF/5-LR cells with SERPINE2 knockdown, showing a significantly increased apoptotic rate compared with control cells. B) Flow cytometric analysis of apoptosis in lenvatinib-treated parental Huh7 cells overexpressing SERPINE2, indicating reduced apoptosis and enhanced drug resistance. C) Immunohistochemical staining of Ki-67 in tumor tissues derived from Huh7-LR xenografts with SERPINE2 knockdown, demonstrating reduced proliferative activity. D) Immunohistochemical staining of Ki-67 in tumor tissues

from PLC/PRF/5 xenografts overexpressing SERPINE2, indicating enhanced tumor cell proliferation.Data are presented as mean ± SD. Statistical analyses were using Student' s t-test or one-way ANOVA as appropriate. Statistical significance is indicated as ns, not significant; *, $p < 0.05$; **, $p < 0.01$; ***, $p < 0.001$.

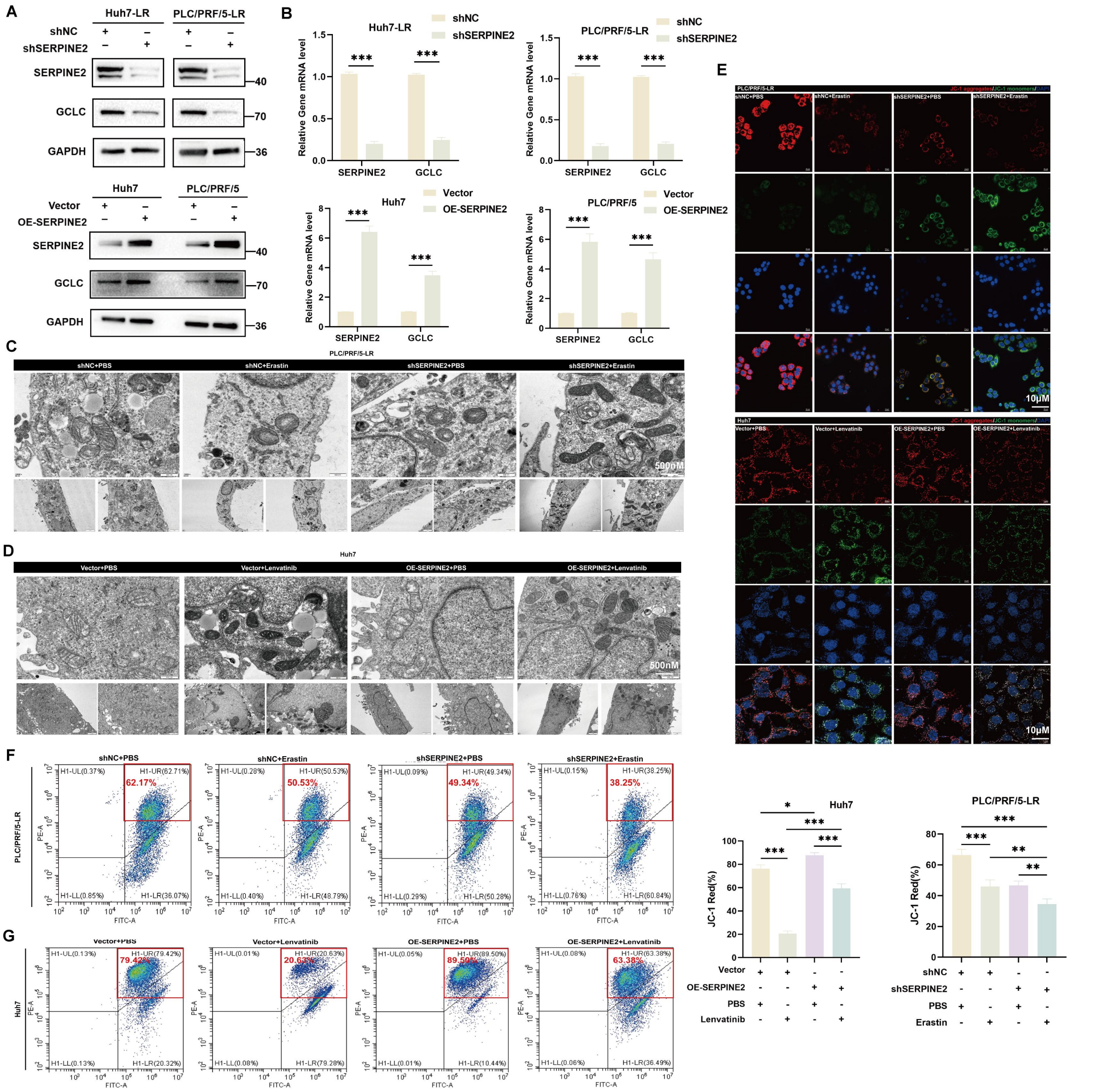

Figure S4. In lenvatinib-resistant hepatocellular carcinoma, there is a critical association between SERPINE2-regulated GCLC and ferroptosis-related pathways, which has been experimentally validated. A-B) Western blot and RT-qPCR analyses validating the regulatory effect of SERPINE2 on GCLC expression. Knockdown of SERPINE2 in lenvatinib-resistant cells markedly reduced GCLC protein(A) and mRNA levels(B), whereas SERPINE2 overexpression in parental cells significantly increased GCLC expression. C) Representative transmission electron microscopy images of mitochondrial ultrastructure in PLC/PRF/5-LR cells treated with erastin, subjected to SERPINE2 knockdown, or receiving

combined treatment, highlighting typical ferroptosis-associated mitochondrial morphological alterations. D) Representative transmission electron microscopy images of mitochondrial ultrastructure in Huh7 cells overexpressing SERPINE2 following lenvatinib treatment, further demonstrating that SERPINE2 overexpression alleviates lenvatinib-induced, ferroptosis-related mitochondrial damage. E – G) Mitochondrial membrane potential changes in Huh7 and PLC/PRF/5-LR cells assessed by JC-1 staining and flow cytometry under conditions of SERPINE2 knockdown or overexpression, before and after treatment with erastin or lenvatinib, supporting a critical role for SERPINE2 in maintaining mitochondrial function and ferroptosis resistance. Data are presented as mean ± SD. Statistical significance was determined using Student ' s t-test or one-way ANOVA, as appropriate. Statistical notations: ns, not significant; *, $p < 0.05$; **, $p < 0.01$; ***, $p < 0.001$.

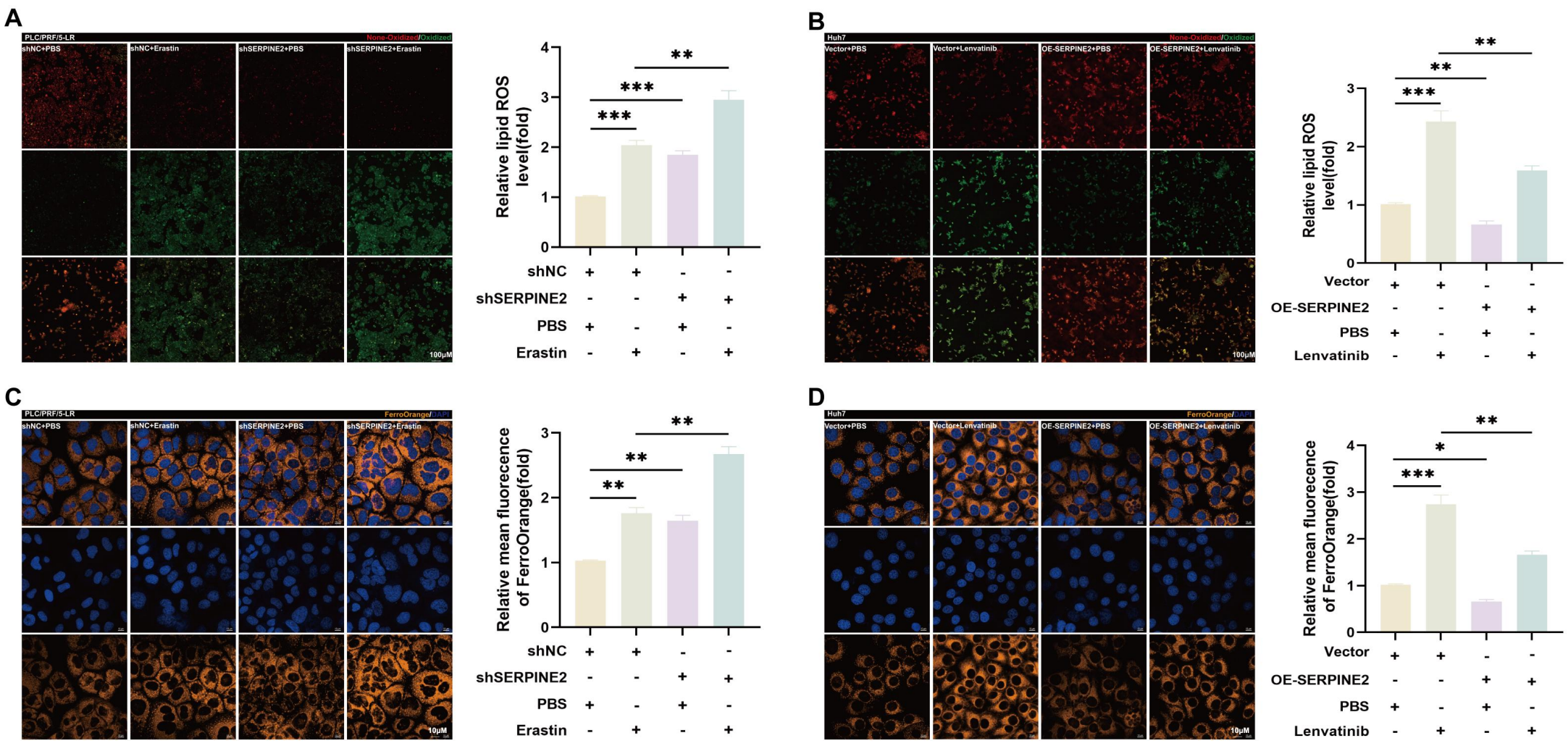

Figure S5. SERPINE2 regulates oxidative stress – related ferroptosis markers in lenvatinib-resistant hepatocellular carcinoma cells. A) Representative confocal fluorescence images and quantitative analysis of lipid reactive oxygen species (ROS) detected by C11-BODIPY staining in SERPINE2-silenced PLC/PRF/5-LR cells treated with or without erastin, showing that SERPINE2 knockdown markedly enhances lipid peroxidation. B) Representative images and quantitative analysis of lipid ROS levels in parental Huh7 cells with or without SERPINE2 overexpression following lenvatinib treatment, indicating that SERPINE2 overexpression alleviates lenvatinib-induced lipid ROS accumulation. C) Quantitative analysis of intracellular ferrous iron (Fe^{2+}) levels in SERPINE2-silenced PLC/PRF/5-LR cells, further supporting that SERPINE2 depletion enhances ferroptotic stress.

D) Analysis of intracellular Fe^{2+} levels in parental Huh7 cells overexpressing SERPINE2 after lenvatinib treatment, showing reduced iron accumulation and enhanced resistance to ferroptosis.Data were analyzed using Student’s t-test or one-way ANOVA as appropriate. Statistical significance is indicated as follows: ns, not significant; *, $p < 0.05$; **, $p < 0.01$; ***, $p < 0.001$.

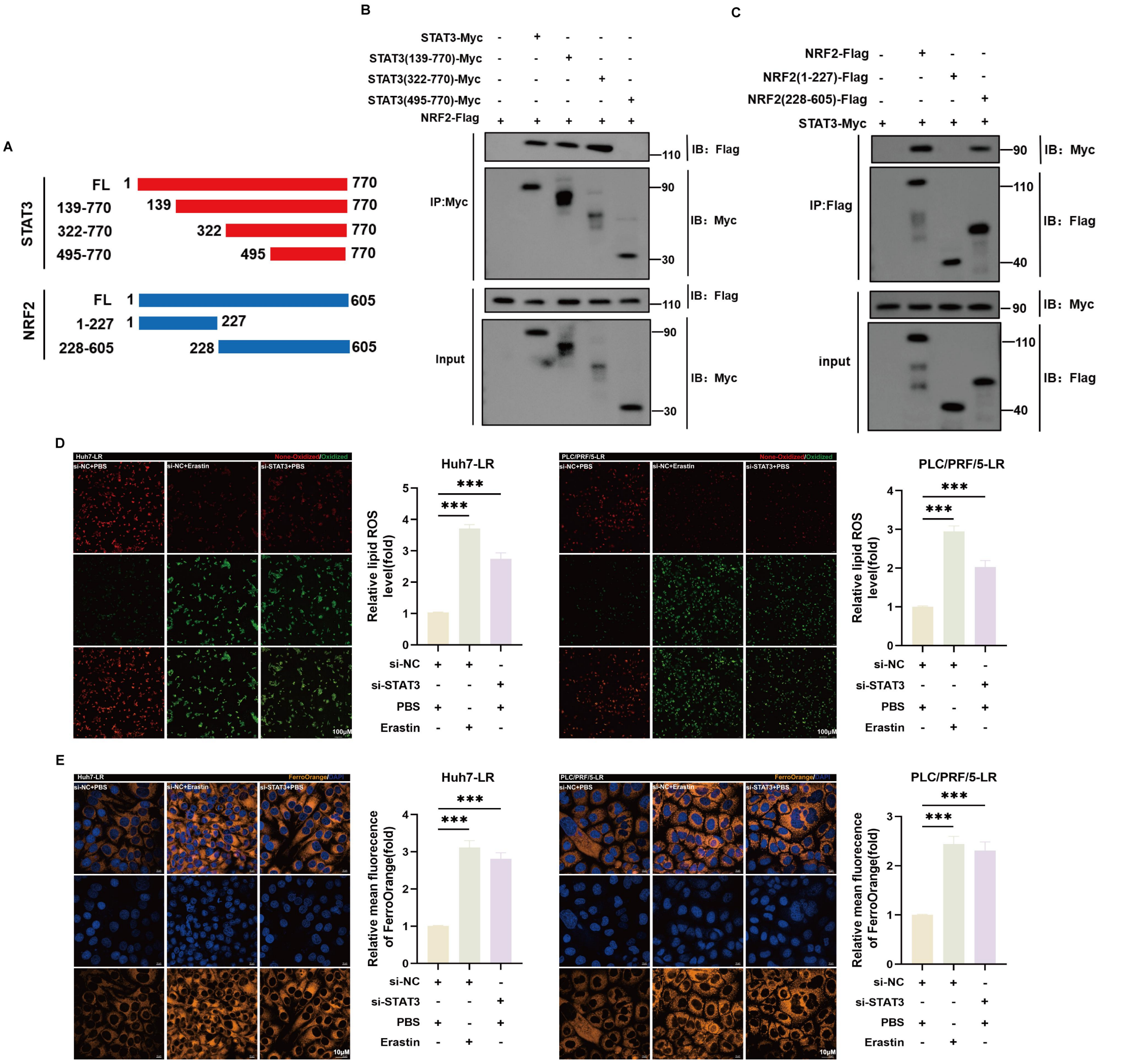

Figure S6. Mapping of the specific interaction domains between STAT3 and NRF2， STAT3 knockdown induces redox imbalance and ferroptosis in lenvatinib-resistant hepatocellular carcinoma cells. A) Schematic illustration of the full-length and serially truncated mutant constructs of STAT3 and NRF2. B)Co-IP mapping assay to identify the precise interaction domain of STAT3. C)Co-IP mapping assay to identify the precise interaction domain of NRF2. D) Representative fluorescence images and quantitative analysis of intracellular lipid reactive oxygen species (lipid ROS) levels detected by C11-BODIPY

staining in Huh7-LR and PLC/PRF/5-LR cells following STAT3 knockdown, showing a significant increase in lipid peroxidation. E) Representative images and quantitative analysis of intracellular ferrous iron (Fe^{2+}) levels detected using the FerroOrange probe in lenvatinib-resistant HCC cells after STAT3 knockdown, demonstrating marked Fe^{2+} accumulation and enhanced ferroptotic stress.Data are presented as mean ± standard deviation (SD). Statistical analyses were performed using Student's t test or one-way analysis of variance (ANOVA). Statistical significance is indicated as follows: ns, not significant; *, $p < 0.05$; **, $p < 0.01$; ***, $p < 0.001$.

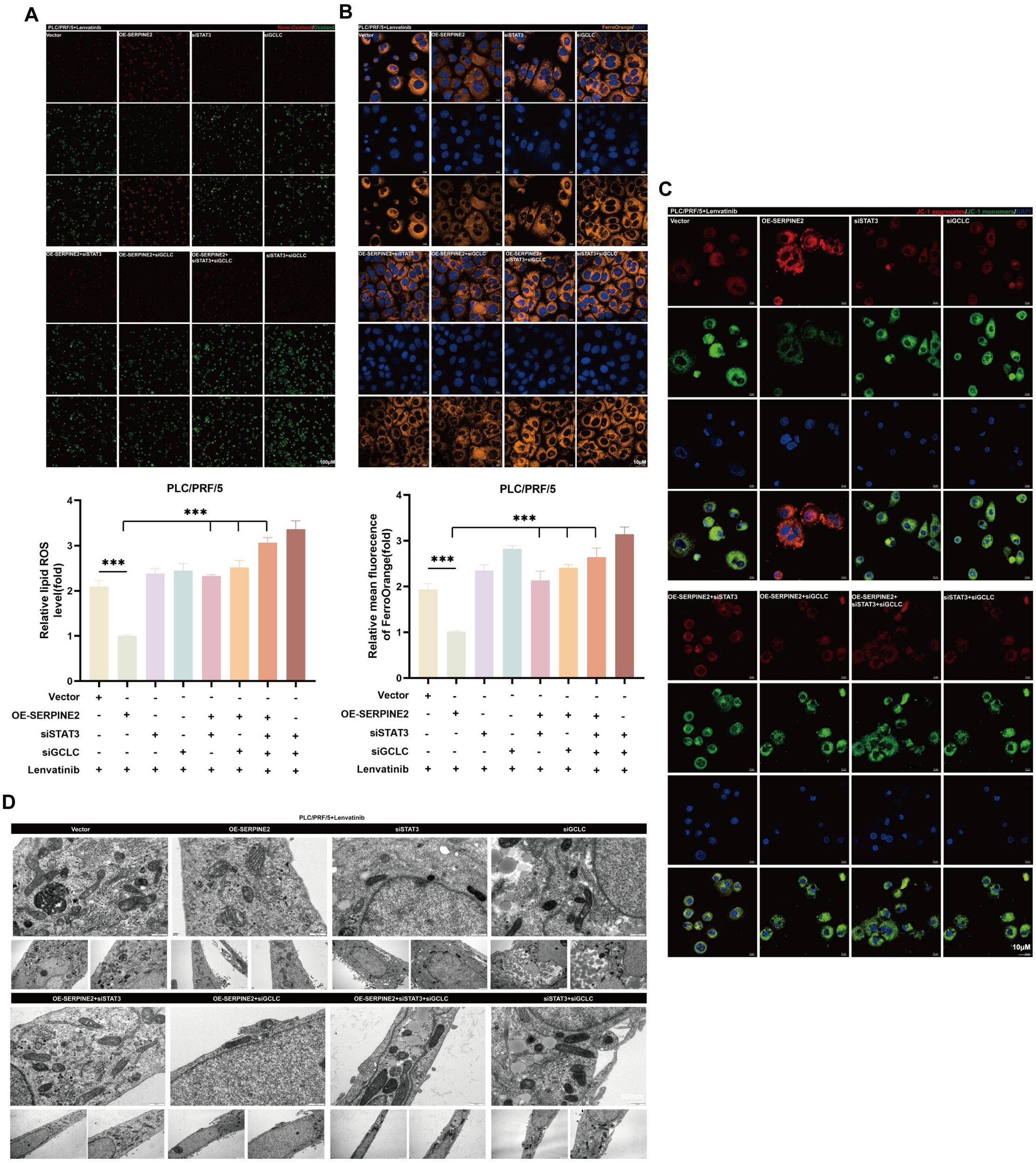

Figure S7. STAT3 or GCLC knockdown reverses the maintenance of redox homeostasis and ferroptosis resistance mediated by SERPINE2. A) In parental HCC PLC/PRF/5 cells

overexpressing SERPINE2, lipid ROS levels were measured by C11-BODIPY staining following STAT3 or GCLC knockdown. Representative fluorescence images and quantitative analysis show that lipid peroxidation was significantly enhanced. B) Under the same conditions, FerroOrange probe was used to detect intracellular Fe^{2+} levels, and representative images and quantitative analysis show that STAT3 or GCLC knockdown significantly promoted Fe^{2+} accumulation. C) JC-1 staining analysis of mitochondrial membrane potential ($\Delta\Psi m$) showed that knockdown of STAT3 or GCLC severely disrupted the mitochondrial function stability maintained by SERPINE2 overexpression. D) Transmission electron microscopy revealed mitochondrial ultrastructural changes. In the context of SERPINE2 overexpression, knockdown of STAT3 or GCLC re-induced typical ferroptosis-associated mitochondrial morphological changes, including mitochondrial shrinkage, reduction or loss of cristae, and membrane structural damage.Data are presented as mean ± SD. Statistical analyses were performed using Student's t test or one-way ANOVA. Statistical significance is indicated as follows: ns, not significant; *, $p < 0.05$; **, $p < 0.01$; ***, $p < 0.001$.

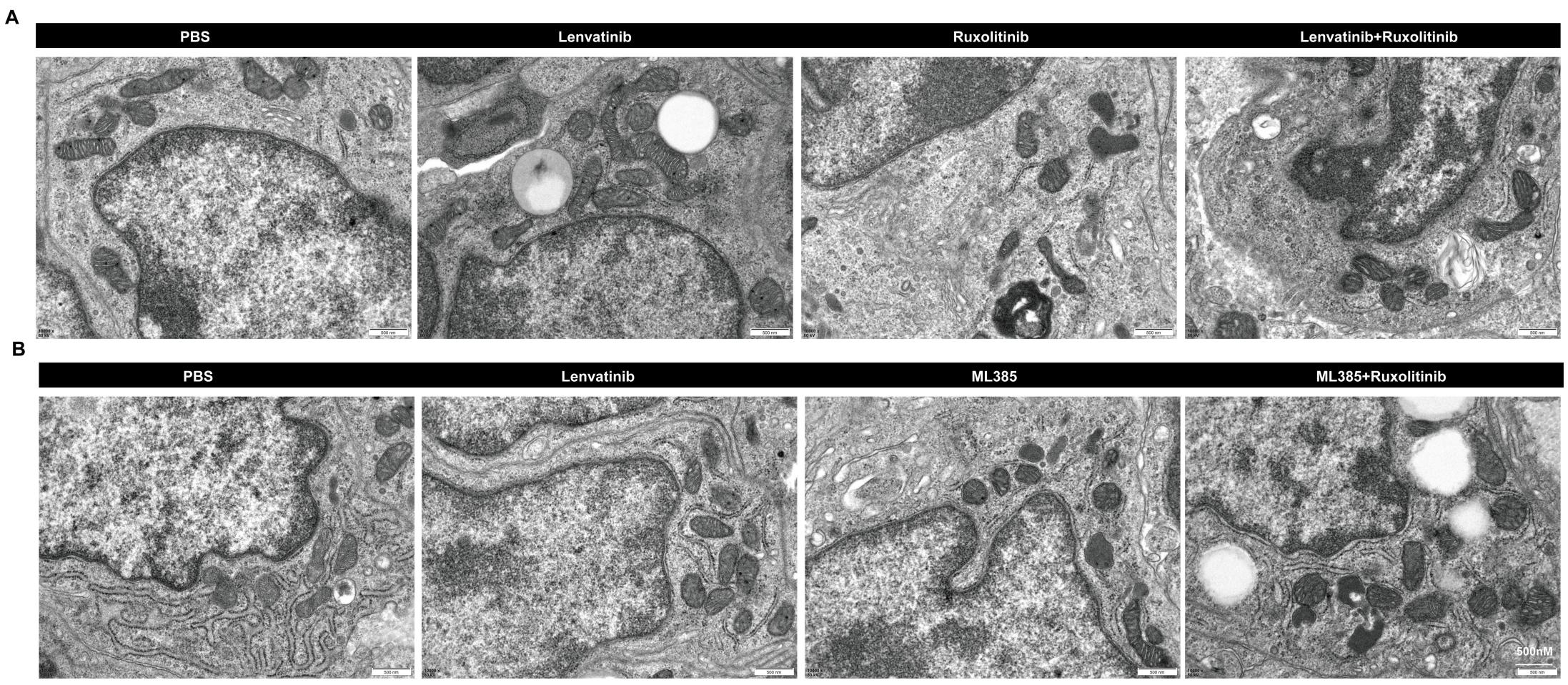

Figure S8. TEM analysis of mitochondrial ultrastructure in orthotopic HCC tumor tissues. A) Representative TEM images of tumor tissues from the orthotopic HCC mouse models treated with PBS, Lenvatinib alone, Ruxolitinib alone, or the combination of Lenvatinib and Ruxolitinib. The combination treatment group exhibits classic ferroptotic mitochondrial alterations, including mitochondrial shrinkage, increased mitochondrial membrane density, and loss of mitochondrial cristae. B) Representative TEM images of tumor tissues from the orthotopic HCC mouse models treated with PBS, Lenvatinib alone, ML385 alone, or the combination of Lenvatinib and ML385. Characteristic ferroptotic mitochondrial

features are predominantly observed in the combination treatment group.
